# Access to hypertension care and services in primary health-care settings in Vietnam: a systematic narrative review of existing literature

**DOI:** 10.1080/16549716.2019.1610253

**Published:** 2019-05-23

**Authors:** Lana Meiqari, Thi-Phuong-Lan Nguyen, Dirk Essink, Marjolein Zweekhorst, Pamela Wright, Fedde Scheele

**Affiliations:** a Athena Institute for Research on Innovation and Communication in Health and Life Sciences, Faculty of Sciences, Vrije Universiteit Amsterdam, Amsterdam, The Netherlands; b Department of Public Health, Institute of Tropical Medicine, Antwerp, Belgium; c Department of Social Medicine, Faculty of Public Health, Thai Nguyen University of Medicine and Pharmacy, Thai Nguyen, Vietnam; d Guelph International Health Consulting, Amsterdam, The Netherlands

**Keywords:** Delivery of health care, hypertension, primary health-care settings, Vietnam, access to care

## Abstract

**Background**: Health care in Vietnam is challenged by a high burden of hypertension (HTN). Since 2000, several interventions were implemented to manage HTN; it is not clear what is the status of patient access to HTN care.

**Objective**: This article aims to perform a systematic narrative review of the available evidence on access to HTN care and services in primary health-care settings in Vietnam.

**Methods**: Search engines were used to identify relevant records of scientific and grey literature. Data from selected articles were analysed using standardised spreadsheets and MaxQDA and following a framework synthesis methodology.

**Results**: There has been increasing interest in research and policy concerning the burden of HTN in Vietnam, covering many aspects of access to treatment at the primary health-care level. Vietnam’s National HTN Programme is managed as a vertical programme and its services integrated into the network of primary health-care facilities across the public sector in selected provinces. The Programme financed population-wide screening campaigns for the early detection of HTN among people above 40 years of age. There was no information on the acceptability of HTN health services, especially regarding the interaction between patients and health professionals. In general, articles reported good availability of medication, but problems in accessing them included: fragmentation and lack of consistency in prescribing medication between different levels and short timespans for dispensing medication at primary health-care facilities. There was limited information related to the cost and economic impact of HTN treatment. Treatment adherence among hypertensive patients based on four studies did not exceed 70%.

**Conclusions**: Although the Vietnamese health-care system has taken steps to accommodate some of the needs of HTN patients, it is crucial to scale-up interventions that allow for regular, systematic, and integrated care, especially at the lowest levels of care.

## Background

### Rationale

The rise of non-communicable diseases (NCDs) and the continued burden of communicable diseases have caused a double burden on low- and middle-income countries (LMICs). According to the ‘Global Burden of Disease Study’ of 2017, NCDs comprised 73% of global deaths [1], with a 40% increase in global ‘Disability-Adjusted Life Years’ [2]. High systolic blood pressure was the main risk factor attributing to ‘Disability-Adjusted Life Years’ [3]. For this reason, high or raised blood pressure, also called hypertension (HTN), is considered a global public health threat with significant economic and social impact [1,4]. At the same time, early detection, adequate treatment and good control of HTN are effective and cost-effective interventions to reduce disability, morbidity and mortality from HTN and its complications such as stroke, ischaemic heart diseases and kidney diseases [1,4–6].

In LMICs, ensuring access to quality HTN care for affected populations is a complex intervention that is better implemented through an integrated primary health-care approach. Such integrated intervention must consider the patient’s health needs for long-term care across time and disciplines which poses significant challenges to the weak health systems and constrained resources in LMICs [4,7].

In Vietnam, a recent Systematic Review and Meta-Analysis showed that the pooled prevalence of measured HTN (i.e. blood pressure ≥140/90 mmHg) was 21% ± 2.6, with lower estimates for the pooled prevalence of those aware of their HTN status (9%) and treated for HTN (5%); these three pooled estimates were significantly lower in rural settings [8]. Since 2008, the Vietnamese Ministry of Health (MoH) implemented several interventions to prevent and manage HTN at the national, provincial, district and commune levels [9]. What remains unclear is the status of patient access to HTN care and services across the primary health-care settings in the Vietnamese health system; synthesising the literature concerning such status would help policymakers and researchers to develop evidence-informed policies, formulate questions for further research, and share lessons learned from Vietnam’s experiences to improve HTN care in resource-constrained settings.

### Objective

This article aims to perform a systematic narrative review of the evidence available in the literature on access to HTN care and services in primary health-care settings in Vietnam. Since this systematic narrative review focuses on the concept of access to care, it follows a framework synthesis methodology [10] utilising the framework on people-centred access to health care proposed by Levenseque et al. [11]. Such methodology is useful in building and consolidating knowledge by accommodating a large number of different types of studies [10].

### Context

The Socialist Republic of Vietnam is a lower-middle-income country with a population of over 90 million, of which 34% is urban [12]. Vietnam has been experiencing demographic and epidemiological transitions. Life expectancy at birth was 76 years in 2016 [13], with a remarkable decline in premature death and disability caused by most communicable, maternal, neonatal and nutritional causes [14,15].

**Figure 1.**
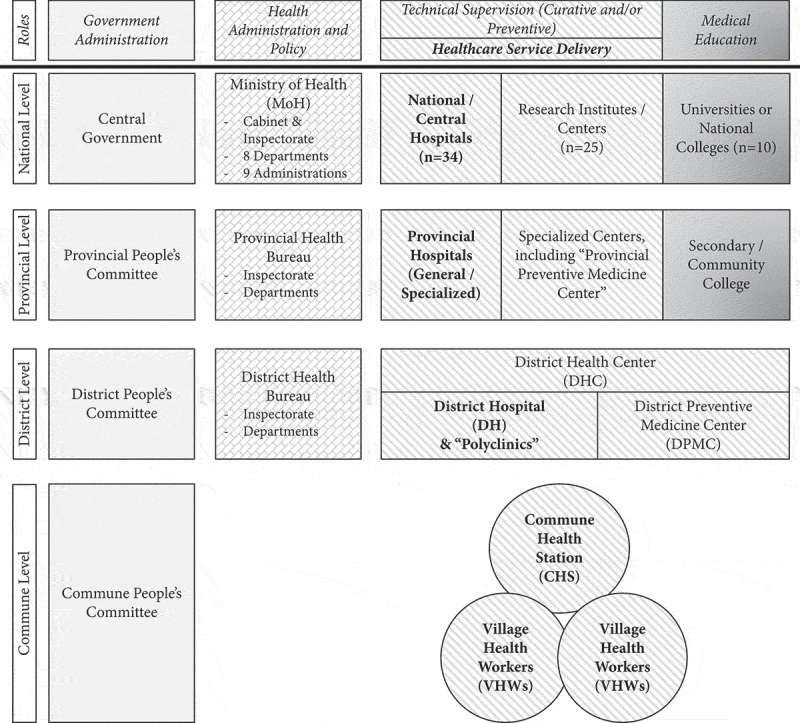
Organisational chart of Vietnam’s health-care system, illustrating roles and responsibilities of each component (adapted from the Ministry of Health).

The health-care system (Figure 1) comprises four levels providing preventive and curative services [16,17]. The commune health station (CHS) is the entry point of care, often comprising a doctor or assistant doctor, a midwife, nurses, an assistant pharmacist and a network of village health workers, and serves a population of 5,000–20,000; CHS team is responsible for providing preventive care programmes, managing common illnesses, providing health counselling, and referring advanced or severe illnesses [16,17]. At the district level, the ‘District Health Bureau’ provides administrative supervision to health services funded by ‘District People’s Committee’, while the ‘District Hospital’ is responsible for managing curative care and ‘District Preventive Medicine Centre’ manages preventive care [16,17]. In some provinces, the last two are integrated into one entity as the ‘District Health Centre’, while in other provinces they operate independently [16,17]. The district facilities provide technical support to the CHSs [16,17]. The commune and district levels are considered to be the grassroots level or the providers of primary health care in Vietnam. The ‘District Hospital’ offers the first referral level of in-patient care [18]. The third level operates at each of the 63 provinces through a ‘Provincial Health Bureau’ or ‘Provincial Department of Health’ which oversees the district and provincial health services, in addition to private health-care facilities [16,17]. The provincial health services include curative care at one general and one or more specialised hospitals and preventive care at the ‘Provincial Preventive Medicine Centre’ [16,17]. The MoH has several departments, national research institutes and national or central hospitals (general and specialised) as the final referral level for therapeutic services [16,17].

The MoH formulates national health policies and national target programmes (NTPs) on health, such as the expanded programme on immunisation and tuberculosis control, most of which are preventive; each NTP focuses on a disease or intervention and is a vertical programme [16,17]. A national hospital, institute or centre manages one NTP and works directly with provincial health entities, which in turn gives direction to district entities to implement the programme at the commune level [16,17]. Central and provincial governments are the primary funders for NTPs; a few of them receive international development assistance [19]. In 2017, the Prime Minister’s office approved the NTPs on health and population for 2016–2020 with eight sub-projects, including the prevention of NCDs [20,21].

Following the adoption of the economic reform plan (i.e. Doi Moi) in 1986, fees were introduced at all levels of the public sector, so public funding covered only part of the medical costs; additionally, the private sector was legalised [22]. Total health expenditure as a percentage of GDP has been consistent at roughly 6% since 2010. In 2014, total health expenditure accounted for 53% of general government expenditure and 46% as private health expenditure [23]. Only 3% of total health expenditure was development assistance for health [23]. Out-of-pocket spending accounted for 37% of total health expenditure and 80% of private health expenditure [23].

The health insurance policy underwent successive changes as shown in Table 1, and recent changes are due to Vietnam’s commitment to achieving universal health coverage [18]. Vietnam Social Security manages the health insurance programme and includes mandatory contributory insurance for formal employees and social health insurance for the rest of the population [18,24]. In 2015, enrolment in social health insurance became compulsory, and the premium was set at 5% of a salary or a minimum wage (~30 USD) [18,24]. For employees, the employer’s contribution is 60% of the total premium [18,24]. The government subsidised the premium for specific population groups, including 100% of the premium for the poor, ethnic minorities, and children under six years of age, at least 70% for the near-poor, and at least 30% for students [18,24]. The insurance coverage by December 2016 was 82% [25].

**Table 1.** Timeline of health insurance and related-government decisions in Vietnam (1986–2016), including health insurance coverage and rate (when available).

1986	Doi Moi Policy for Economic Reforms
1987	No changes
1988	No changes
1989	Pilot for first Voluntary Health Insurance (VHI)
	Introducing user fees, private sector
1990	No changes
1991	*Health Insurance Coverage Population*: Children <6yrs
1992	Introducing Social Health Insurance (SHI) under Vietnam Health Insurance Agency (VHIA)
	*Health Insurance Coverage Population (Rate*): Civil servants, employees in state and non-state (>10 employees) enterprises, pensioners, elderly (4%)
1993	No changes
1994	Introducing fee exception scheme for poor
	*Health Insurance Coverage Population*: Poor in addition to schools and kindergartens
1995	Introducing schemes of Health Insurance Fund (HIF) under Vietnam Social Security (VSS), including VHI
1996	No changes
1997	No changes
1998	Introducing 20% co-payment, special conditions for special groups
	*Health Insurance Coverage Population (Rate*): PLUS: Government members, preschool teachers, social welfare target groups, dependants of policy and armed forces staff (23%)
1999	Introducing nation-wide fee exemption scheme for poor
2000	Defining health insurance as a non-life insurance business sub-sector
2001	No changes
2002	Introducing Health Care Fund for the Poor (HCFP)
	Decentralising and budgetary autonomy to local provincial agencies
2003	Merging VHIA into VSS; Encouraging VHI by introducing a minimum threshold 30% for total uninsured at the community level
2004	Law on Protection, Care and Education of Children
2005	Implementing the medical insurance regulation; Detailing payment methods: fee for service and training, and prepare for case-based (diagnosis-specific) package payment mechanism; Introducing VHI in every commune with no co-payment and VSS is implementation agency
	*Health Insurance Coverage Population (Rate*): PLUS: Non-state (>1 employee) enterprises, cooperatives, other organisations, war veterans, poor (46%)
2006	Expansion of accountability and rights of public hospitals
2007	Raising contribution rates of VHI; Increasing premiums of individuals enrolled in VHI; Transferring the responsibility of some health insurance duties to MoH
	Local administrations cannot raise taxes without legal grounds and act on their sole discretion; Developing Vietnam’s pharmaceutical industry; Introducing first law on personal income tax; Implementation guidelines for the Law on Insurance Business (issued in 2000), however, it did not touch on health insurance business activities
2008	Increasing premiums of individuals enrolled in VHI; At least 50% of government subsidies on health insurance premiums to enrol in the VHI scheme for members of ethnic groups, social-welfare targeted, poor and near-poor households
	Investing in construction, improvement, upgrading of district-level general hospital for period 2008–2010
2009	Health Insurance Law/Law on Health Insurance
	*Health Insurance Coverage Population (Rate*): PLUS: Children <6yr, near poor (60%)
2010	*Health Insurance Coverage Population (Rate)*: PLUS: Students (65%)
2011	No changes
2012	*Health Insurance Coverage Population (Rate)*: PLUS: Farmers (66%)
2013	Accelerating the implementation of health insurance policies and legislation for universal health coverage
	Regulations on division of medical care by level for health-care facilities
2014	Revised Health Insurance Law/Law on Health Insurance (amendment and supplementation of several articles)/Amended Health Insurance Law
	*Health Insurance Coverage Population (Rate)*: PLUS: Dependants of labourers and cooperative members; Compulsory participation by 2016 (70%)
2015	Introducing a roadmap to develop and implement basic health service package paid by health insurance in Vietnam
2016	*Health Insurance Coverage Rate*: 82%

## Methods

The reporting of this review follows the ‘Preferred Reporting Items for Systematic Reviews and Meta-Analyses’ (PRISMA) statement [26], as relevant to a narrative and descriptive analysis rather than a quantitative and statistical one.

### Conceptual framework

**Figure 2.**
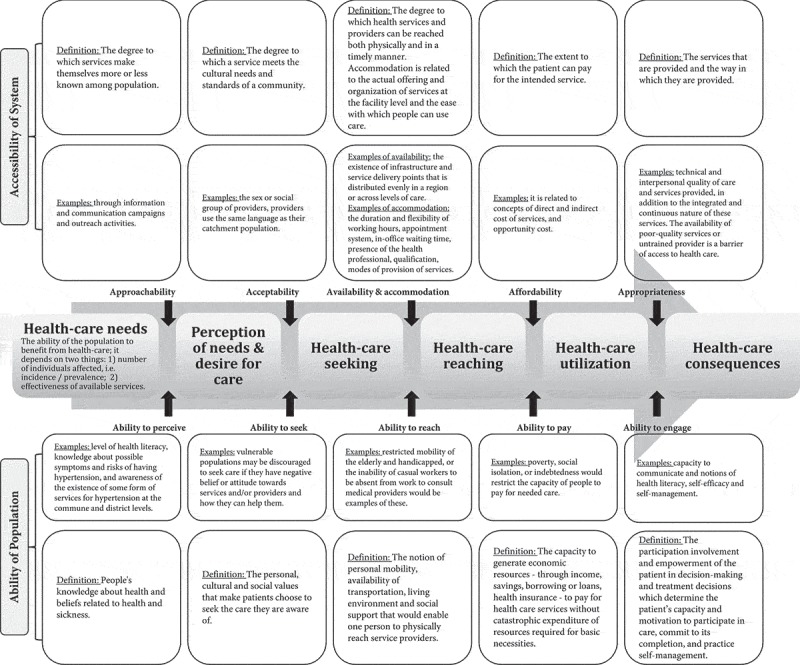
Framework on people-centred access to health care with definitions and examples for each component, modified from Levenseque [24].

This review uses the framework on people-centred access to health care proposed by Levenseque et al. [11] to develop search terms, the analysis plan, and the synthesis of the results. Access and other related concepts such as accessibility, availability, and utilisation are usually used interchangeably [27]. This article will use the definition of access as ‘*the opportunity to identify health-care needs, to seek health-care services, to reach, to obtain or use health-care services and to actually have the need for services fulfilled*’ [11]. Levenseque *et al*. described access as a multidimensional concept resulting from the interaction between five dimensions of accessibility for the health system that provides or supplies care (namely, *approachability, acceptability, availability and accommodation, affordability, and appropriateness*) and five corresponding abilities of populations who demand care (namely, *ability to perceive, ability to seek, ability to reach, ability to pay, and ability to engage*) [11]. These characteristics are defined in Figure 2. For example, a person suffering from symptoms related to HTN (e.g. a continuous headache and nosebleeds) should be able to realise one’s health needs and act upon them by seeking health services in the public or private sector. Furthermore, people should be able to reach these health services (e.g. located nearby) and use them (e.g. pay for services without consequences). In turn, the health system should be ready to provide appropriate care to meet the patient’s needs (e.g. measure blood pressure to give the correct diagnosis and course of action).

Furthermore, Levenseque *et al*. argued that these characteristics are not independent, but they interact and influence each other differently during an episode of illness and care [11]. For people living with HTN, their health-care needs include continuity of care defined as ‘*the provision of coordinated care and services over time and across levels and disciplines, which is coherent with the patient’s health needs and personal circumstances*’ [28]. The need for continuity of care is reflected in the framework by examining the activation of the access process by an empowered patient beyond the first contact, as it is relevant each time the patient attempts to access care [11].

### Eligibility criteria

We developed eligibility criteria following the elements of population, concept, and context. The population under study are people living with HTN in Vietnam, which excludes Vietnamese refugees and migrants living abroad. The concept is the broad aspects of access to health care and services as defined by the conceptual framework. The context is settings which provide primary health care in Vietnam; this includes grassroots level (i.e. district and communal levels) and out-patient clinics at the provincial level, in addition to private clinics. The search included any existing scientific and grey literature; written in English; both quantitative and qualitative; with no limits to the type of study design, such as empirical studies, systematic reviews, meta-analyses, letters, guidelines and policy documents. The period under focus was 2000 until the search date (6 June 2017).

### Information sources and search strategy

The following databases were searched: PubMed, EMBASE, CINAHL, and Web of Science, in addition to grey literature identified through Google, and the websites of WHO, other United Nations agencies, and the Vietnamese MoH. The search process included: (a) Initial limited search for one online database to analyse the text words contained in the title and abstract of retrieved records, and the index terms used to describe them; (b) Second search using all identified keywords and index terms across all included databases; and (c) Snowball retrieval of texts using the reference list of all identified records looking for additional ones. Detailed tables on the search terms used are provided in S1 Table.

### Selection process

One researcher (LM) screened the title and abstract using the Rayyan QCRI application [29]. Since one researcher was available to perform the selection process, a quality check was performed by two researchers (DE and MZ) independently cross-checking a random selection of 10 articles which resulted in full agreement over the screening results [30]. Then, full texts were obtained and read to assess each record’s eligibility; this was conducted twice within one month to compare the results between readings at two separate time points [30].

### Data extraction

LM used a standardised spreadsheet to extract relevant data including author, publication year, objective, project of the study, data collection year, location, population and sampling, disease/s under investigation, relevant outcome measure/s, key findings, key discussion points, reported limitations, reported future steps, and reviewer’s notes on quality and relevance of the article. Then, articles were exported to MaxQDA and codded deductively based on themes identified by the conceptual framework, and inductively to add emerging themes. The use of inductive thematic analysis matches a more robust strategy of a single researcher conducting data extraction at two separate time points [30].

### Data analysis and synthesis

The process of framework synthesis included analysing and integrating the data within each theme and performing a cross-case synthesis for the included articles to compose the findings [31]. This review aims to summarise qualitative and quantitative evidence narratively. Thus, quality assessment and heterogeneity were explored descriptively during data extraction, analysis and synthesis [31]. Additionally, relevant policy documents and decisions written in Vietnamese were reviewed by TPLN for cross-checking and further explanations.

## Results

### Study selection

**Figure 3.**
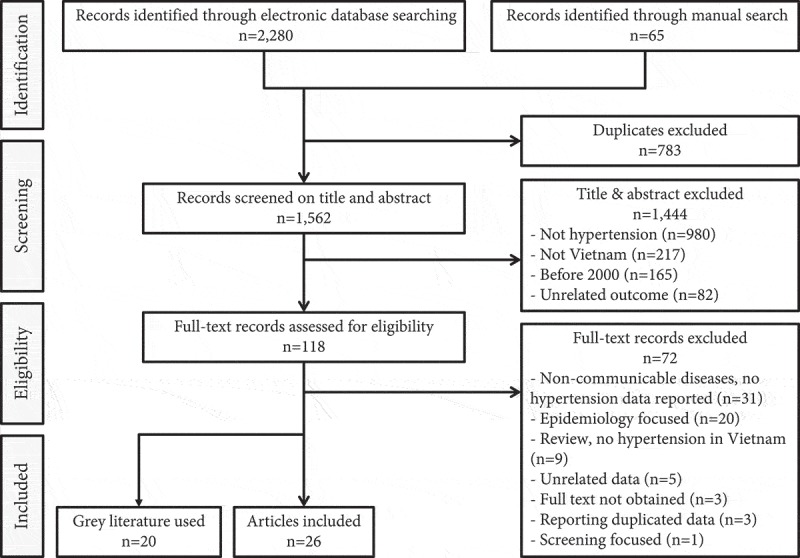
Flow diagram for study selection process.

There were 2,280 records identified from the database search and 65 from the manual search (Figure 3). Of these, 783 were duplicates, and 1,444 were excluded based on ‘title and abstract’ screening. The full texts of the remaining 118 records were examined, of which 26 peer-reviewed articles and 20 records of grey literature were included in this review. The grey literature included official documents published by the Vietnamese MoH and United Nations agencies, in addition to doctoral dissertations (note: five peer-reviewed articles used data from three doctoral dissertations).

### Study characteristics

An overview of each article is presented in Tables 2 and 3 divided as peer-reviewed articles and grey literature documents. Almost half of the studies investigated HTN as a primary outcome, while most of the system’s peer-reviewed articles and official documents had information on HTN as part of NCDs or their risk factors. For the 26 peer-reviewed articles, 13 (50%) described dimensions related to the accessibility for the health system [32–44], 10 (39%) described dimensions related to the abilities of the population to access care [45–54], and 3 (11%) on the MoH intervention to control HTN which described dimensions of both domains [55–57]. The cross-sectional design was the most used one among peer-reviewed articles (n = 10; 39%), the majority of which looked at aspects of the abilities of the population (n = 7).

**Table 2.** Characteristics of included peer-reviewed articles.

No.	First author	Objective	Population	Sampling technique	Sample size
**Accessibility of system**					
	**Cross-sectional**				
1.	*Tuan, 2005*	To gather evidence about the availability and quality of the community health system in general, and private health services in particular	Health centres in one commune	Multistage stratified cluster	CHSs: n = 30 HC providers: n = 126 Private providers: n = 234
2.	*Mendis, 2012*	To evaluate the capacity of primary care to implement basic interventions for prevention and management of major NCDs, including CVDs and diabetes	Health centres in one district	Random	n = 15
3.	*Hoang, 2014*	To describe the primary care system in a selected rural area in Vietnam in terms of its current capacity for the prevention and control of chronic NCDs; collecting data on the current status of the six building blocks of the primary care system.	Health centres in one district	All centres	Districts centres: n = 2 CHSs: n = 18
	**Literature review**				
4.	*Hoang, 2009*	To report and discuss currently available evidence on economic aspects of chronic diseases in Vietnam	NA	NA	NA
5.	*Alwan, 2010*	To review the capacity of countries to respond to NCDs	NA	NA	NA
6.	*Abdul Rahman, 2015*	To discuss the growing problem of hypertension in the Asia-Pacific region, and to develop consensus recommendations to promote standards of care across the region	NA	NA	NA
	**Intervention**				
8.	*Barzin, 2012*	To investigate a charity’s medical programme focusing on its impact on the public health system	Patients & HC providers	Non-random clinic recruitment	NS
9.	*Sundberg, 2012*	To describe an education and training programme for health practitioners in Vietnam on prescribing physical activity	HC providers	NS	NS
10.	*Islam, 2014*	Summary from a talk in the Symposium titles as: Health systems and NCDs in developing countries: experience from Vietnam	HC providers	NA	NA
11.	*Markuns, 2015*	To develop systems to effectively train, support and integrate competent primary care physicians for health systems as part of an effort to address human resource development of primary care staff.	HC providers	NA	NA
12.	*Do HTP, 2016*	To evaluate the effectiveness of the Eat Less Salt (ELS) intervention with a view to scaling up to a regional or national level	Adults aged 25–64 yr	Screening (non-random)	Baseline: n = 509
				Follow-up after 1yr	Follow-Up: n = 511
	**Therapeutic randomised trial**				
13.	*Nguyen MH, 2012*	To investigate and examine the effects of Tai Chi on physical fitness, perceived health, blood pressure, and preventing falls among the elderly	Community-dwelling elderly 60–79 yr	Patients recruited, randomly divided to intervention & control groups	Intervention: n = 39 Control: n = 34
14.	*Nguyen HL, 2017*	To report the results of a cluster-randomised feasibility trial at three months follow-up conducted in Hung Yen province, designed to evaluate the feasibility and acceptability of two community-based interventions to improve hypertension control: a ‘storytelling’ intervention, ‘We Talk about Our Hypertension,’ and a didactic intervention	Adults ≥50yr living in 4 communes	Cluster randomisation Screening for all adults invited	n = 331
			hypertensive patients	Follow-up after 3mo	Storytelling: n = 79 Didactic: n = 80
**Ministry of Health (MoH) intervention**					
15.	*Nguyen QN, 2011*	To summarise our approaches on how to implement a programme on hypertension management in rural commune in Vietnam, and to involve all related partners, and finding potential factors which could influence local people’s adherence	Community-based study Adults ≥25yr in one commune	Random	n = 1,180
			hypertensive patients	Follow-up after 17mo	n = 469
16.	*Nguyen QN, 2012*	To evaluate the impact of healthy lifestyle promotion campaign on CVD risk factors in the general population in the context of a community-based programme on hypertension management	Quasi-experimental study Adults ≥25yr in two communes	Random in each commune (baseline vs. 3yr follow-up)	Intervention: n = 1,131 vs. n = 1,185 Reference: n = 1,162 vs. n = 1,190
17.	*Lim, 2014*	To examine cases of innovation and identify critical success factors in NCD management in ASEAN	Review	NA	NA
**Ability of population**					
	**Cross-sectional**				
18.	*Duong, 2003*	To determine the risks associated with hypertension in Vietnamese communities around Ho Chi Minh City	Adults living in one city	Non-random screening	n = 357
19.	*Son, 2012*	To characterise the prevalence and distribution of hypertension, together with awareness, treatment and control in the general adult population (25 years and over) in Vietnam, with a view to providing a better evidence base for health planning.	Adults ≥25yr nationally	Multistage stratified cluster	n = 9,823
20.	*Boas, 2012*	To study the prevalence of undiagnosed hypertension and the treatment of those diagnosed with hypertension	Adults aged ≥35yr in 6 communes	Randomised cluster	n = 1,621
21.	*Ha DA, 2013*	To describe the prevalence, awareness, treatment, and control of hypertension, and to examine factors associated with these among the adult population residing in Thai Nguyen province, a mountainous northern region of Vietnam	Adults ≥25yr in one province	Multistage stratified cluster	n = 2,348
22.	*Ha NT, 2014*	To measure quality of life among hypertensive people in a rural community in Vietnam, and its association with socio-demographic characteristics and factors related to treatment	hypertensive patients >50yr managed in one CHS	Random	n = 275
23.	*Do HTP, 2015*	To present the national prevalence of pre-hypertension and hypertension and their determinants, as well as levels of awareness, treatment, and control, based on a large nationally representative sample of Vietnamese adults examined in 2005	Adults 25–64 yr nationally	Multistage stratified cluster	n = 17,199
24.	*Nguyen TPL, 2015*	To evaluate differences in health-state utilities related to characteristics of these patients to identify potential predictors using the Short-form 36 version 2TM (SF-36v2) questionnaire to collect data on health-related quality of life (HRQoL)	hypertensive patients <80yr	Non-random clinic recruitment	n = 691
	**Modelling**				
25.	*Nguyen QN, 2012*	To estimate the time trends in blood pressure, body mass index (BMI), and smoking status in adults Vietnamese population over a nine-year period and highlight the differences between men and women as well as the differences between urban and rural areas	Adults 25-74 yr nationally	5 cross-sectional surveys (2 included in review)	n = 23,563
	**Qualitative**				
26.	*Veith, 2016*	To identify the critical barriers facing patients with hypertension when trying to access care	hypertensive patients Physicians	Non-random clinic recruitment	Patients: n = 89 Physicians: n = 3
	**Cohort**				
27.	*Nguyen TPL, 2017*	To (i) assess the level of adherence of hypertensive patients visiting CHSs in a rural area in Vietnam; (ii) examine the relationship between level of adherence and cardiovascular risk among hypertensive patients; and (iii) get a better understanding of adherence and factors influencing adherence among these patients	Adults aged 35–64 yr in 4 communes	Random	n = 3,779
			hypertensive patients	Follow-up for 1yr	Survey: n = 315 In-depth interviews: n = 18

NCDs, non-communicable diseases; CVDs, cardiovascular diseases; CHSs, commune health stations; HC, health care. NA: Not applicable; NS: Not specified.

**Table 3.** Characteristics of included grey literature documents.

No.	First author, Year	Title	Publisher
	**Joint Annual Health Review (JAHR**): Vietnam Ministry of Health (MoH) & Health Partnership Group		
1.	2007	JAHR 2007	Vietnam MoH & Health Partnership Group
2.	2008	JAHR 2008: Health financing in Viet Nam	Vietnam MoH & Health Partnership Group
3.	2009	JAHR 2009: Human Resources for Health in Vietnam	Vietnam MoH & Health Partnership Group
4.	2010	JAHR 2010: Vietnam’s health system on the threshold of the five-year plan 2011–2015	Vietnam MoH & Health Partnership Group
5.	2011	JAHR 2011: Strengthening management capacity and reforming health financing to implement the five-year health sector plan 2011–2015	Vietnam MoH & Health Partnership Group
6.	2012	JAHR 2012: Improving quality of medical services	Vietnam MoH & Health Partnership Group
7.	2013	JAHR 2013: Towards universal health coverage	Vietnam MoH & Health Partnership Group
8.	2014	JAHR 2014: Strengthening prevention and control of non-communicable diseases	Vietnam MoH & Health Partnership Group
9.	2015	JAHR 2015: Strengthening primary health care at the grassroots towards universal health coverage	Vietnam MoH & Health Partnership Group
	**More official documents**		
10.	Harper C, 2011	Vietnam noncommunicable disease prevention and control program 2002–2010: Implementation review	World Health Organization (WHO)
11.	2012	One Plan 2012–2016 between the government of the Socialist Republic of Viet Nam and the United Nations in Viet Nam	
12.	2014	Guidelines for diagnosis, treatment, prevention of hypertension: 1^st^ Vietnam Congress of Hypertension	Vietnamese Society of Hypertension & Vietnam Heart Association, 2014
13.	2016	Five-year Plan: For people’s health protection, care and promotion in the period 2016–2020	Vietnam MoH
14.	2016	Independent Review of the One Plan (2012–2016) between the Government of the Socialist Republic of Viet Nam and the United Nations in Viet Nam	SIPU
15.	2016	National survey on the risk factors of non-communicable diseases (STEPS) Viet Nam, 2015	Vietnam MoH & WHO
	**Doctoral dissertation**		
16.	Hoang VM, 2006	Epidemiology of cardiovascular disease in rural Vietnam	Umea University: Sweden
17.	Nguyen QN, 2012	Understanding and Managing Cardiovascular Disease Risk Factors in Vietnam: Integrating Clinical and Public Health Perspectives	Umea University: Sweden
18.	Son PT, 2013	Hypertension in Vietnam: From community-based studies to a national target programme	Umea University: Sweden
19.	Duong DB, 2015	Understanding the service availability for non-communicable diseases prevention and control at public primary care centres in Northern Vietnam	Harvard Medical School: USA
20.	Nguyen TPL, 2016	Health economics of screening for hypertension in Vietnam	University of Groningen: The Netherlands

**Figure 4.**
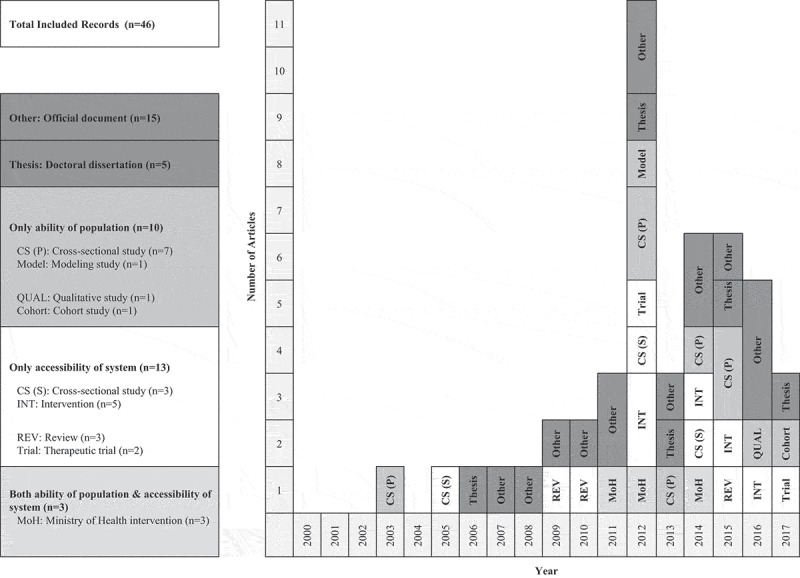
Timeline for included studies and records by publication year, framework and type (n = 46) Population; ability of population. System; accessibility of system. MoH; Ministry of Health.

Figure 4 shows a timeline for the selected records by their publication date. The number of records published increased over time, especially after 2010. In 2012, the number of records peaked (n = 11, 24%) and articles with a primary focus on HTN became more frequent. Geographically, data on study sites were available for 17 peer-reviewed articles (65%) and two doctoral dissertations unrepresented in peer-reviewed articles. Almost 75% of study sites were located in two northern regions of Vietnam. No individual study was conducted in Central Highlands.

### Analytical framework and themes

**Figure 5.**
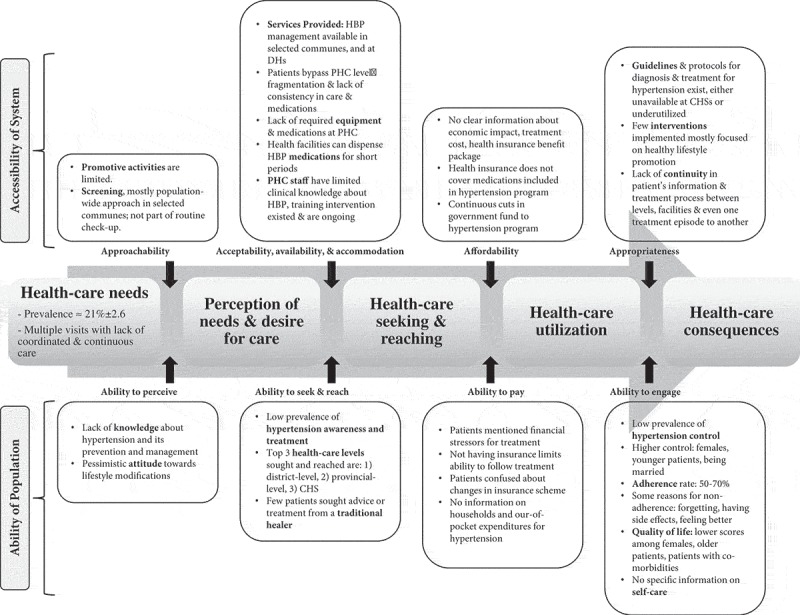
Overview of key results on access to hypertension care at primary health care in Vietnam based on the framework on people-centred access to health care. HBP, high blood pressure; DHs, district hospitals; PHC, primary health care; CHS, commune health station.

Results are presented below under five main themes: one emerged theme on governance and policy, and four themes are based on the conceptual framework (i.e. perception of needs and desire for care, a merged theme for health-care seeking and reaching, health-care utilisation, and health-care consequences). For each theme, results are summarised under the relevant dimensions for accessibility for the health system and the abilities of the population to access care. Key findings of the conceptual framework are illustrated in Figure 5.

## Governance and policy

The National HTN Programme was established in 2008 as part of the NTP for the prevention and control of NCDs which contains five vertical or disease-specific projects for HTN, diabetes, cancer, asthma and chronic obstructive pulmonary diseases, and mental and neurological disorders. The National HTN Programme is managed by the Vietnam National Heart Institute at Bach Mai Hospital in Hanoi under the direction of the MoH; the primary objectives of this Programme for the period 2012–2015 can be summarised in two main categories [9]:

1. Promoting public knowledge about HTN. As part of these efforts, the government declared 15 May as a nationwide ‘Hypertension Day’, and created a local website to provide information and resources about HTN for both the public and health professionals [22].
2. Sustaining a model for HTN management at the grassroots level to treat patients according to guidelines. The National HTN Programme piloted the model at selected CHSs in 16 provinces, then expanded to 32 provinces, and finally to all provinces. The model included conducting population-wide screening for early detection at the commune level, and HTN being managed at CHSs with referrals as appropriate; additionally, health workers in implementing communes were trained on management and treatment of HTN [22].

In 2015, 10% of communes nationwide implemented the HTN management model [18], which did not cover all CHSs due to a reported lack of resources [9,58]. The National HTN Programme received funding in 2010, two years after its establishment [9]. Since 2012, the NTP budget, which comes from central and provincial governments, has been declining [18]. There has been limited external funding for NCD programmes [9]. For example, one of the aims of the ‘United Nations’ One Plan’ (2013–2016) was to improve evidence on effective and efficient prevention and management of NCDs at national and sub-national levels [59]; an independent review reported this goal to be only partially achieved due to lack of funding [60].

In March 2015, the Government of Vietnam approved the national strategy for the prevention and control of NCDs for the 2015–2025 period; one of its goals is to scale up NCDs management models, including HTN, to cover 90% of health facilities at the primary level (i.e. district and commune) [18]. However, according to the 2015 Joint Annual Health Review (JAHR), the ‘Strategy for the Protection, Care and Promotion of the People’s Health for the period 2011–2020ʹ, which is the roadmap used to create the goals of the five-year health-sector plan, remains focused mainly on infectious diseases [18]. In general, most of the latest Vietnamese government documents and policies mention the challenges of both NCDs and infectious diseases as priorities for the health system [18,20,61].

## Perception of needs and desire for care

### System’s approachability

Despite the increase in promotional activities to educate the public about NCDs and their risk factors, these prevention efforts remain ‘*fragmented, lack professionalism, and have limited effectiveness*’ [62]. Both district and commune levels were sometimes engaged in conducting campaigns to reduce tobacco use, salt intake and alcohol consumption, and the district level considered more active than commune level. However, activities to promote physical exercise and sports were rare at any level [34]; this was explained at the JAHR 2011 by the ‘*lack of guidance for practice, appropriate methods, in addition to lack of practice facilities and sites*’ [62].

The National HTN Programme also supported and financed community-outreach campaigns following a population-wide screening model for the early detection of HTN among people aged 40 years and above; this model could not cover the entire population as it was organised periodically as a campaign at selected CHSs [9,34]. On the other hand, opportunistic screening, i.e. routine blood pressure check-ups during any patient’s visit to CHSs, is not yet integrated at the commune level due to underfunding [9,16]. Ideally, MoH and researchers recommended a combination of population-wide and opportunistic screening [9,35,63].

### Population’s ability to perceive

In Vietnam, the proportion of adults aged 18–69 years old who did not attend formal schooling or did not finish primary school was 17% according to a 2015 survey [64]. Generally, there is still a lack of knowledge and awareness about HTN among the general population and patients, including risks, disease status, prevention, and management [38,45,53,65]. A 2013 survey on HTN-related knowledge found [65] ‘*a high level of basic knowledge, but a low level of specific HTN knowledge*’. Furthermore, people with more basic knowledge about HTN were significantly found to be living in urban areas, better educated, had checked their blood pressure within a year, or had a previous diagnosis of HTN. The primary source of information reported was mass media (e.g. television, national/local radio, and newspapers).

People also have limited knowledge about lifestyle modifications to prevent and control HTN and its risk factors [45,48], and hypertensive patients were reported to have a pessimistic attitude towards such modifications [53]. For example, a 2013 survey to detect HTN in Thai Nguyen asked participants if they ‘must have salt when eating’ [48]. Among people with HTN, those unaware of their condition were less aware of the risks of salty food than patients aware of their disease; the adjusted odds ratio was not significant. Among people with HTN aware of their status, those treated were less informed about the risks associated with eating salty food than untreated patients (odds ratio = 2.7; 95% CI: 1.4–5.4), which means that being treated did not increase patients’ odds of knowing about the risks of eating salty food [48].

## Health-care seeking and reaching

### The system’s acceptability, availability, and accommodation

There was no information on the acceptability of health services for HTN care and management, especially regarding the interaction between patients and providers. Information on the availability of resources in health centres has reported their limited capacity to provide care for HTN, especially in CHSs located closer to patients [34,62], and the contribution of the private sector was also described as limited [34]. Patients, therefore, tended to bypass the commune level and go to more distant centres, which increases their costs [34]; this impedes continuous support for disease management. Moreover, HTN management at district and commune levels is based mainly on measuring blood pressure and rarely takes into account behavioural or metabolic risk factors (e.g. smoking, total blood cholesterol, and the presence or absence of diabetes mellitus) [9]. Three studies described the existing capacity of CHSs for NCDs, including HTN [32–34] and three sub-themes emerged from the analysis of these studies: basic equipment, medication, and human resources.

#### A. Basic equipment

There is variation in the availability of basic equipment at different levels and sectors of the health system [32,33,62]. The MoH regulations about required equipment in a CHS that has a doctor include a device to measure blood pressure (i.e. sphygmomanometer) and a simple biochemistry testing machine and urinalysis [9]. A 2003 survey found that most CHSs had at least one functional sphygmomanometer with a stethoscope; however, the proportion of private clinics with either item was 63% and 73%, respectively [32]. Furthermore, a capacity assessment conducted in one district in 2011 reported that essential blood and urine tests (i.e. blood glucometer, urine ketones test strips, and urine protein test strips) were not available in most CHSs, and patients had to be referred to higher levels or the private sector [34].

#### B. Medication

Both public and private sectors had NCDs medication available [32,36]. Table 4 shows a drug list for HTN control comparing drugs’ availability based on the MoH Health Insurance Drug Formulary and two assessments of CHSs [9,33,34]. The table shows that three types of HTN medication were intermittently available at CHSs: ACE inhibitors, calcium channel blockers, and thiazide diuretics, in addition to aspirin. Although beta-blockers, loop diuretics, and statins might be available in some CHSs, health insurance does not cover them at commune level.

**Table 4.** Availability of selected essential hypertension (oral) medication based on health insurance drug formulary and two studies that investigated capacity of commune health centres.

Selected essential HTN medicines	Health Insurance Drug Formulary, 2011		Health Insurance Drug Formulary, 2014	Commune Level Document, 2017	*Mendis, 2012* *(n = 15)*	*Van Minh, 2014* *(n = 18)*
	District Level	Commune Level	When different from previous edition	When different from Health Insurance Drug Formulary		
**Diuretics**						
**Thiazide**						
*Hydrochlorothiazide*, %	+	+			*40.0*	*100.0*
Indapamide	+	−				
**Loop**						
*Frusemide*^a^, %	+	+			*86.7*	*61.1*
**Potassium sparing**						
*Spironolactone*^a^	+	+				*0.0*
**Adrenoceptor antagonists**						
**β-blockers^b^**						
*Atenolol*^c^, %	+	+			*93.3*	*16.7*
Acebutolol, Bisoprolol, Carvedilol, Metoprolol, Nebivolol	+	−				
Labetalol	+	−	+ Commune Level			
Propranolol^c^	+	+				
**α1-selective blocker**						
Doxazosin	+	−				
**Centrally acting α_2_-agonist**						
Clonidine	+	+		−		
Methyldopa	+	+				
**Centrally acting I_1_-selective imidazoline receptor agonist**						
Moxonidine, Rilmenidine	+	−				
**Calcium-channel blockers**						
**Dihydropyridine**						
*Amlodipine*, Nifedipine, %	+	+			*86.7*	*66.7*
Cilnidipine, Felodipine, Lacidipine, Lercanidipine, Nicardipine	+	−				
**Phenylalkylamine**						
Verapamil^d^	+	+				
**Benzothiazepine**						
Diltiazem^c^	+	−				
**Targeting renin-angiotensin system**						
**Angiotensin-converting enzyme (ACE) inhibitors**						
*Enalapril*, Captopril, %	+	+		+	*60.0*	*61.1*
Perindopril	+	+		−		
Benazepril Hydrocloride, Imidapril, Lisinopril, Quinapril, Ramipril	+	−				
**Angiotensin II receptor antagonist**						
Losartan	+	−	+ Commune Level			
Candesartan, Irbesartan, Telmisartan, Valsartan	+	−				
**Direct Arterial Vasodilators**						
Hydralazine	+	−	- District Level			
**Combined medication**						
**Thiazide diuretic with beta-blocker**						
Hydroclorothiazide + Bisoprolol	+	−				
**Thiazide diuretic with ACE inhibitor**						
Indapamide + Perindopril	+	+		−		
**Thiazide diuretic with angiotensin II receptor antagonist**						
Hydroclorothiazide + Losartan	+	−	+ Commune Level	−		
Hydroclorothiazide + one of (Irbesartan, Telmisartan, Valsartan)	+	−				
**ACE inhibitor with calcium-channel blocker**						
Perindopril + Amlodipine	+	−				
**Other**						
*Isosorbide dinitrate*^c^, %	+	+			*60.0*	*0.0*
**Statin, lipid-lowering medication**						
Atorvastatin	+	−	+ Commune Level	+		
Fenofibrat^d^	+	+				
*Simvastatin*^d^, %	+	−	+ Commune Level	+	*46.7*	*0.0*
*Acetylsalicylic acid (Aspirin)*, %	+	+			*100.0*	*55.6*

(+), available; (−), not available Commune-level document, Vietnamese official document with list of medications that should be available at commune health stations. *Italic*, medication investigated by empirical studies. NA, medication is not reported in the study, although according to Health Insurance Drug Formulary it can be available at commune and district levels.

^a^ Medicines are listed with diuretic drugs and not hypertension drugs.

^b^ According to 2014 Joint Annual Health Report, beta blocker is not reimbursed at commune level, because it should be provided by the National Hypertension Program.

^c^ Medicines are listed with antianginal drugs and not hypertension drugs.

^d^ Medicines are listed with Lipid-lowering agents.

Patients seeking medication in the public sector faced two problems. First, there is fragmentation and lack of consistency in prescribing medication between different levels [9]. For example, doctors at higher levels may prescribe newer-generation medication that is not covered by health insurance at CHSs; if the patients want to keep using the same medication, they have to return to the higher-level facilities or purchase them at their own expense [9]. While most basic HTN medication is cheap, newer generations may be less affordable [32,36]. Second, current regulations, according to JAHR 2014, allow provincial facilities to dispense HTN medication for 15 days, district facilities for 7–10 days, and CHSs for 5–7 days [9]; in practice, provincial and district hospitals dispense HTN medication for 30–45 days. These short periods of prescribed medication require more visits to health facilities, which increases the treatment cost for patients, reduces their compliance, and decreases the odds of HTN control.

#### C. Human resources

In the public sector, there are different types of health worker at CHSs, including doctors, midwives, assistant physicians, an assistant pharmacist and nurses [32,34]. In the private sector, one commune’s assessment showed that 11% of those working at private clinics had no official medical qualifications or certificates, and approximately 80% are practising without registration [32]. Traditional practitioners and pharmacists accounted for 20% of the private workforce; also, almost 37% of CHS staff had a private practice [32].

The level of clinical knowledge about HTN management is limited in both public and private sectors [16,32]. One assessment from 2003 asked providers ‘what questions they would raise with a 58-year-old man presenting with high blood pressure?’, then calculated a score for each provider as the percentage of mentioned items out of 11 expected correct answers. Most respondents scored poorly; only two public providers could specify ≥70% of correct answers, while other providers identified <50%, including 70% of public providers (n = 30), 80% of private–public (n = 53), and 90% of private providers (n = 129) [32]. Another survey from 2012 showed that the proportion of CHSs staff who knew how to diagnose HTN was 51%; while in a self-assessment survey among newly qualified doctors, only 38% could monitor and manage chronic diseases in the community [66].

Several opportunities are still ongoing to train health workers at district and commune levels on the prevention and management of HTN [39–41]. For example, the MoH Decision number 1816, dated 2008, stated the responsibility of staff from higher-level facilities to support staff from lower-level facilities by providing in-service training [19]. Also, the National HTN Programme conducted training of trainers for doctors from all provinces; these provincial trainers then taught medical or nursing staff at the district and commune levels; however, in most provinces, the training has not yet covered all centres [9]. Village health workers had limited access to training, although they were reported to be involved in the early detection and monitoring of HTN cases [34]. Available details about training projects are in S2 Table.

### Population’s ability to seek and reach health care

Findings from different surveys showed a low level of HTN awareness and treatment. For example, the latest national survey on risk factors for NCDs reported that 30% of the study population aged between 18 and 69 years had never had their blood pressure measured by any health worker [67]. Higher odds of people with HTN being aware of their disease status were reported among women [46–48,67], the oldest age group [46,48,67], a higher level of education [46,48] and having a family history of HTN [46]. The prevalence of treatment among HTN patients according to five studies ranged between 12% and 30% [46–48,50,67]; the treatment prevalence was higher among those aware of their condition [46,48,50]. Higher odds of treatment were found among men [63], urban residents [46,47], a lower level of education, and having a family history of HTN [46]. According to one study, people of ethnic minorities were more likely to be aware of their HTN status and less likely to be on treatment [48].

Only 14% of people with HTN reported having their disease managed at a health facility according to the 2015 national survey on risk factors for NCDs [67]. Patients often described the resources at the CHSs or public sector in general as sub-standard [38,53], and the top three health-care levels sought and reached were district level (34%), provincial level (27%), and CHSs (19%) [67]. Other reported options were central hospitals, the private sector, traditional healers, and self-treatment [65,67]. However, there was not enough information in the literature regarding the use of private clinics, traditional healers, drug stores and pharmacies.

## Health-care utilisation

### System’s affordability

There is limited information related to the cost and economic impact of treating HTN. No economic evaluation was performed for the MoH intervention, although the manager reported in a review that patients continued to pay for the medicines out of their own pockets after the end of the funding for HTN medicines [57]. One study estimated the cost of HTN treatment at the CHS level to be 70.8 USD per person per year, almost 6 USD per person per month [68]. On the other hand, a simulation model estimated that scaling up an HTN programme to cover 80% of patients could avert 266,000 deaths over the period 2005–2006 [35]. Such a programme would include providing treatment for high-risk individuals with aspirin and drugs to lower blood pressure and cholesterol; this treatment, in turn, would reduce disease progression, prevent complications such as heart attacks and strokes, and reduce complication expenses [35]. It is unclear if such a scaling up is possible given the continuous cuts in government funding for the National HTN Programme. The funding for the NCDs programme was cut by 68% in 2014 compared to 2013 [9]. In 2013, the total funds for NTPs accounted for nearly 70 million USD, of which 13% was allocated to the NCDs programme, and only 17% of NCDs programme funds went for HTN (nearly 1.5 million USD). At the grassroots level, there is no budget allocated explicitly for the prevention, management and treatment of NCDs [18,33,34]; the primary sources of funding for each district are the state budget through the Provincial Department of Health, user fees and health insurance based on a fee-for-service model [34]. While CHSs should receive a minimum of 10% of the health insurance fund allocated to the district level, they were often allocated precisely this amount, which was not enough to meet the cost of medical needs for people with NCDs at the commune level [9].

There are different types of health insurance coverage. Some patients may have 100% coverage for all health services, and others may have a 20% co-payment [22]. One of the goals of the Law on Health Insurance was to create a mechanism for CHSs and district facilities to provide primary medical services and to make them more accessible to people at the grassroots [66]. The 2014 revision of the Law on Health Insurance states that only curative care services can be reimbursed, such as patients who have symptoms and visit health facilities to get their blood pressure measured and medication in line with the MoH Health Insurance Drug Formulary; however, no preventive care services are covered, including population-based screening for early detection [9]. Subsequent implementation guidelines have not specified details for HTN care, such as a list of interventions covered by the health insurance fund [18]. In 2015, the MoH published a roadmap towards developing and implementing a basic health service package paid by health insurance in Vietnam [61], a pilot study for which is ongoing.

For years, health insurance did not reimburse types of HTN medication included in the National HTN Programme budget (e.g. beta blockers), even if the programme’s distribution was limited. The cuts to the national fund for NCD programmes have further reduced its ability to procure required medical supplies; however, the health insurance policy was not adjusted to compensate for this reduction, and patients needed to pay for their medication [9,57]. In such cases, patients might not purchase medication or even a full or regular dose [19].

Furthermore, the limited scope of services and medication available and covered by the health insurance scheme at the CHS level have in many cases caused patients to bypass the CHSs to higher levels [34]. At the district level, NCD patients who have health insurance received free treatment provided that CHSs referred them; patients who have insurance but who bypass the CHSs have to pay for full treatment costs [34]. Patients might even bypass the entire primary health-care system to the secondary level (i.e. provincial hospitals); this leads to higher out-of-pocket payments through direct and indirect costs (e.g. co-payments that are higher at secondary and tertiary facilities and costs of transport and accommodation) [40,66].

### Population’s ability to pay

There was limited information about the association of HTN management with economic or working status. One article reported a higher risk of being hypertensive among wealthier men and poorer women [35,69]. However, there was a non-significant association between having health insurance and a patient’s ability to follow treatment; the MoH found that hypertensive subjects without insurance did not join its intervention (odds ratio = 1.4; 95% Confidence Interval is 0.8–2.4) [55].

There is no specific information on health expenditure for HTN. However, hypertensive subjects mentioned financial barriers to obtaining care, including financial stress and confusion about changes in the insurance scheme [53]. On the other hand, some financial information was available for a general category of chronic diseases or NCDs showing catastrophic health expenditure for most households [34,35]; these information includes diseases such as cancer with expensive treatments and is not appropriate estimations when discussing HTN.

## Health-care consequences

### System’s appropriateness

The National HTN Programme/MoH issued the protocol for diagnosis and treatment of HTN in 2010 and disseminated it widely [9]. The latest version of this protocol was approved in 2013. However, at CHSs, the HTN protocols, the WHO/International Society for HTN risk prediction charts, and other essential guidelines for health education and counselling on risk factors and early detection of NCDs were either unavailable or underused [34]. Also, health facilities could apply other local guidelines only if they were published officially, for example, the annual recommendations of Vietnam Association of Cardiology regarding the treatment options for hypertensive subjects based on the stage of their disease [37,70]. The main difference between these two documents was in the level of detail in the information presented, with no conflict in their contents.

There is not enough information to evaluate the appropriateness of treatment decisions and services provided by the health system. One study investigated the nature of treatment for HTN patients [47] and reported that the vast majority of those aware of their HTN (n = 212, 91%) had been recommended to modify their behaviour (e.g. taking exercise, reducing sodium or salt intake, giving up smoking, or losing weight). Besides, the most common medication used by 60% of the hypertensive subjects included calcium channel blockers (n = 54; 59%) and ACE inhibitors (n = 36; 39%).

Most articles discussing hypertension have recommended the need to implement both a population-wide strategy (e.g. salt reduction) and a patient-focused clinical or behavioural strategy to treat HTN and avoid its complications [35,42–46,50,56]; these included two interventions: the MoH intervention and Eat Less Salt intervention [42,56]. Both interventions included healthy lifestyle campaigns promoting behavioural change to lower blood pressure and improve HTN control [42,56]. The results from both interventions showed a significant reduction in systolic and diastolic blood pressure and a less salty diet in the general population [42,56]. However, the MoH intervention reported limited effects on smoking and alcohol consumption [56].

Lack of continuity in the health system was extensively reported as a significant barrier to accessing high quality and affordable medical services, especially for chronic diseases. Many factors contribute to this lack of continuity. For example, at the administrative level, there is a separation between curative and preventive services, especially at the district level. This separation is more pronounced in the provinces that followed the MoH Decree in 2008 on splitting the District Health Centre, which provided integrated curative and preventive services and directly managed CHSs in two different entities (i.e. District Hospital and District Preventive Medicine Centre) [71]. Additionally, at the clinical level, patients face discontinuity in their medical information and treatment process between levels, facilities, and even from one treatment episode to another, given the lack of organised referral/back-referral system to track their progress [19]. Besides, providers at higher level hospitals often practise overprovision of curative care services leading to an increased disconnect between levels [18]. Mendis’ assessment reported that using multidisciplinary teams was not feasible or affordable at CHSs level, and there was no organised appointment/reminder system for people with HTN to come for follow-up [33]. However, in Dong Hy district’s assessment, communes within the WHO project or national NCD programme (n = 7/18) mentioned implementing active and continuous management and treatment of HTN, with no details about the mechanism used to provide long-term management and continuity of care [34]. To facilitate information sharing, a software package to manage records of hypertensive subjects was established in 36 provinces across the health system’s levels and facilities, in which health workers can enter hypertensive patients’ information after each visit to the CHS. However, data extracted are used only to send reports to a higher level of care, and health insurance for reimbursement, and not for treatment follow-up [9].

### Population’s ability to engage

Relevant findings included information on the prevalence of HTN control, treatment adherence, and quality of life; there was no specific information on self-care or self-management practices. There was a low prevalence of hypertensive patients with controlled blood pressure; the prevalence ranged between 3% and 16% [46–48,50,67]. Among treated patients, nearly a third had controlled blood pressure [46,48,50]. The gap between the prevalence of HTN and the prevalence of treatment and control persisted between 2001 and 2009 even though the prevalence of treatment and control have increased significantly over time; this meant that a high proportion of people with HTN were undiagnosed or untreated, which could result in higher stroke-related mortality [54]. Higher odds of control were found among younger women and married individuals [46].

The prevalence of treatment adherence among hypertensive patients based on four studies did not exceed 70% and was even less than 50% in a one cohort study [44,49,52,55]; however, the definition of adherence differed across the studies (see S4 Table). Programme compliance in the MoH intervention with three-year follow-up was independently associated with changes in systolic or diastolic blood pressure measurements and the number of anti-hypertensive drugs prescribed [55]. The head of the programme reported that continuous follow-up and management encouraged patients to continue taking the medicines, even after the end of the funding [57]. The cohort study [52] used a qualitative inquiry to further investigate the reasons for adherence and non-adherence among selected patients. In many cases, patients classified as non-adherent in a quantitative analysis of facility-based data mentioned during interviews that they tend to buy their medicines from pharmacies rather than getting them from the CHS. The main factors leading to good adherence were either awareness of possible complications of high blood pressure or experience of such complications within a patient’s family. The two main reasons for not taking HTN medication were forgetting or experiencing side-effects. Also, two patients actively decided not to take the pills when they felt better.

Finally, two studies measured the quality of life among hypertensive subjects using different scales [49,51]; however, they identified similar factors associated with a lower score of quality of life, including older patients, women, and those with co-morbidities. An intervention study investigated the effect of Tai Chi exercise programme on the health of older adults including their quality of life; findings showed that the intervention group had a significant improvement in their perceived health, which remained for at least eight weeks after training ended [43].

## Discussion

There has been increased interest in research and policy concerning the burden of HTN in Vietnam, which covered many aspects of access to HTN care at the primary health-care level. In general, Vietnam’s National HTN Programme is managed as a vertical programme with its services integrated into the public network of primary health-care facilities in selected provinces [72]. The scale-up of the National HTN Programme has occurred gradually; however, fewer than half of CHSs have implemented activities for HTN [25]. Also, the programme is facing continuous budget cuts. While the treatment of HTN is integrated into the primary health-care services, there is still a lack of integration between levels of care and with other vertical programmes for NCDs which share similar risk factors and their long-term nature. Collaborative efforts with different policy sectors (such as national NCDs programmes, preventive programmes and the insurance programme) are needed to overcome barriers to prevention and control services for NCDs including HTN at the health-care system, health-care provider and patient levels [73]; these efforts should take into account the context of Vietnamese society and its health system. Many barriers in access to HTN care are similar to what is reported in other international studies and settings, such as low patient health literacy; overburdened health-care providers; the lack of an organisational structure to accommodate a non-physician as a primary care provider; the lack of confidence and/or policy towards the non-physician providers’ ability to manage uncomplicated and stable patients; the lack of infrastructure for data collection and monitoring of clinical information on a periodic basis; and finally, limited resources [73,74]. Generally, new policies need to leverage existing resources with limited ones assigned for overall health care and NCDs. Our discussion will focus on a number of key aspects: (1) screening; (2) acceptability; (3) accommodation of medication and treatment guidelines; (4) health-care use; (5) long-term nature of HTN disease and care including adherence, self-management and quality of care; (6) task shifting; and (7) the role of the private health sector.

First, population-wide screening has been essential to the National HTN Programme strategy for prevention and control of HTN. A review of systematic reviews published in 2015 found insufficient evidence to confirm a beneficial effect of blanket screening for HTN and/or diabetes in LMICs compared to other types of screening methods; the authors urged caution regarding the implementation of population-based mass screening, which mobilises scarce resources without adequate evidence of its effects [75]. Studies from Vietnam mentioned the importance of population-wide screening in both health promotion and secondary prevention, but also called for including opportunistic check-ups for high-risk groups at all health facilities. A policy statement made by the World HTN League called for implementing systematic approaches to the screening of blood pressure by having a routine assessment at each clinical encounter, regardless of the complaints that led the patient to visit the clinic [76]. An analysis of data on persons aged 50 years or older from six LMICs estimated that just one year of routine opportunistic HTN screening during formal visits to health-care providers could yield significant increases in HTN awareness among older people in developing countries; this opportunistic screening added the advantage of leveraging existing resources and infrastructure, as well as facilitating a direct transition from the point of diagnosis to subsequent expert counselling and clinical care for newly identified HTN patients [77]. At the same time, the effectiveness of all screening programmes (i.e. community-outreach or opportunistic during clinic visits) depends on having a proper mechanism to refer people with high blood pressure for further diagnosis and management [76–78].

Second, research from other countries identified acceptability as an essential factor for access to HTN care. Case studies from South Africa on barriers to health care for the chronically ill reported that the necessary elements for strengthening the public sector not only require better drug supply chains, ambulance services, referral systems and clinical capacity and addressing the financial barriers, but also need to consider how providers can engage with patients to strengthen the therapeutic alliance [79].

Third, results have shown the limited accommodation of HTN medication at the primary health-care level in Vietnam, in addition to patients’ confusion regarding whether and how the costs of the medication are covered by health insurance. Patients reported using alternative sources for medication such as private drug stores and pharmacies; however, there is no specific information on accessibility to these alternative sources, especially on out-of-pocket expenditures. Participants in a study to explore individual and community preferences for a health benefit package among the uninsured in Vietnam identified both costly services that are seldom used (e.g. in-patient care) and less costly services used on a frequent basis (e.g. drugs and out-patient care) as a priority in the design of any health benefit package; the participants justified their wish to cover prescribed medication through health insurance because of the cost associated with this benefit and its vital role in treatment [80]. WHO’s recommendations for HTN care in low-resource settings include the availability of a core set of essential anti-hypertensive medication in primary health-care facilities that have a doctor on the staff: thiazide diuretic, calcium channel blocker (amlodipine), beta-blocker (atenolol), and angiotensin inhibitor (enalapril), in addition to medication used in presence of other risk factors for cardio-vascular condition or strokes, for example, statin (simvastatin) and aspirin [7]. Furthermore, policies must consider each patient’s need for continuous access to these medications with a reasonable timespan for their repeated visits to the health services. Although Vietnam has standardised treatment guidelines, they should be contextualised and aligned with the health insurance policy and the availability of medication at different levels of care.

Fourth, there is scarce evidence regarding the nature of the use of HTN care and services in Vietnam. People mentioned being confused about the health insurance scheme. These results are similar to a survey from 2011 on public knowledge and understanding of health insurance schemes and their benefits, in which 30% of heads of household did not know about health insurance, and 92% did not know or gave the wrong answer for the premium rate [81]. However, there is no information on out-of-pocket payments for HTN care, especially given that many patients reported buying medication from private pharmacies. Available data for a general category of NCDs found that people with chronic conditions spend 19% more on out-patient services than those with other health conditions [82]. Further analysis should consider the impact of repeated visits to lower and higher levels of care, which are expected to cause extra costs.

Fifth, as LMICs are facing an emerging burden of NCDs, there are increased global calls on the role of strengthening primary health care and ensuring universal health coverage in their prevention and control, especially to achieve a challenging need for NCDs patients represented by the continuity of care [83–85]. Continuity of care is aimed to provide patients with coherent life-long care in three essential dimensions: longitudinal care, the nature of the patient–provider relationship, and coordinated care across levels and disciplines [86]. Given that many LMICs, especially in sub-Saharan Africa, have experienced continuity of care through HIV programmes, many practical examples of successful HIV interventions were proposed for possible extension to NCDs such as care delivered with a family focus; paper-based or electronic appointment systems for scheduled follow-up; on-site medical records; mobile phone applications or SMS reminders; use of peer educators and patient groups for counselling and adherence support; standardised treatment protocols; and task shifting and task sharing [87,88]. Such interventions must be considered by research and policy to establish and strengthen the continuity of care that can promote patients’ adherence to treatment and build their skills in self-management and self-care, which eventually help in achieving better health outcomes.

Sixth, recent evidence stressed the effectiveness, cost-effectiveness, and efficiency of task-shifting interventions at primary health-care facilities, including community health workers [74,89,90]. The success of such interventions is likely to depend on context (e.g. demographic factors and the characteristics of community or village health workers, care settings, and training programmes). Policy and research should, therefore, focus on the possibility of contextualising task-shifting interventions in Vietnam’s health system.

Seventh, questions have been raised regarding the role of drug stores and pharmacies in access to medication, and subsequently, adherence to treatment, as an additional option to leverage all existing resources towards better HTN care. For example, research from Tanzania looked at the feasibility and acceptability of screening for HTN in private drug retail outlets [91] and found that out of 971 customers, only one refused to be screened. However, only 20% of those newly diagnosed visited the referral clinic within two weeks. Another qualitative study from Indonesia looked at the role of community pharmacists in the management of chronic NCDs [92]. The findings described a positive impact on the regular interactions between patients and community pharmacists as their care providers driven by mutual recognition of each other’s roles and their ability to communicate. However, the research called for systematic support from different stakeholders to improve the primary care practice of community pharmacists towards further continuity of care. Vietnam could also investigate and build on the role of the private health sector, including clinics, pharmacies, drug stores, and traditional medicine practitioners.

Finally, essential information is needed to craft evidence-based policies towards facilitating access to HTN care in Vietnam; information about the impact of key social determinants of health (e.g. ethnicity), perception of needs and desire for care (i.e. acceptability of services and the ability of the population to seek and reach care), including the patient’s attitude towards providers and available care, in addition to the provider’s motivation to care for patients, especially for those in need of long-term care as hypertensive patients are. Other critical factors are the development of a basic benefits package for health insurance, in addition to assessing health-care use specifically for HTN patients and dimensions of the continuity of care (e.g. longitudinal care and coordinated care including information management). Some aspects of access and continuity of care need further qualitative research, such as the interaction and nature of the relationship between patients and providers.

### Limitations

First, our literature search has not explicitly incorporated information available only in the Vietnamese language such as articles published in Vietnamese journals, or doctorate and master’s theses written for Vietnamese Universities. However, JAHR is expected to include most good-quality studies published in Vietnamese and we incorporated official documents in their original language. As a next step, it is important to conduct a separate review into Vietnamese literature which also aims to investigate the differences and similarities between such articles and those published in international journals in terms of both methods and results. Second, we did not undertake a full quality assessment of the identified articles since our review included all eligible articles rather than focusing on a subset of studies. Yet, the subsequent analysis of included articles and official documents found no contradictory results or information [93].

## Conclusion

Although the Vietnamese health-care system has taken steps to accommodate some of the needs of HTN patients, it is crucial to scale-up interventions that allow for regular, systematic, and integrated care, especially at the lowest levels of care. Therefore, a system to allow the commune level to properly receive back-referred patients and monitor stable ones should be established and strengthened; such asystem is vital to facilitate patients’ adherence to treatmentand continuity of care. Also, this monitoring systemshould be supported with appropriate resource allocations which in turn strengthen approaches for primary health care and universal health coverage.

## Paper context

In limited-resources settings, health systems are facing an increased burden of chronic conditions (for example, hypertension in Vietnam). Vietnam is facing a high burden of hypertension. Services to patients are integrated into primary health-care network. Despite progress, challenges arise with the system’s approachability, accommodation, and appropriateness. People need support in perceiving health needs, seeking, reaching and engaging with care. Commune levels need further support to provide regular and systematic care and follow-up to stable patients.
