# Supplementary material for: Access to hypertension care and services in primary health-care settings in Vietnam: a systematic narrative review of existing literature
Source: Glob Health Action. 2019 May 23;12(1):1610253. doi: 10.1080/16549716.2019.1610253 (PMC6534204; doi:10.1080/16549716.2019.1610253)
Supplement: Supplemental Material [file ZGHA_A_1610253_SM1717.zip › S Table 1.docx]

**Search terms and strategy performed in four databases (i.e. PubMed, EMBASE, CINAHL, and Web of Science)**

**PubMed**

| **No.** | **Query** | **6/6/2017** |
| --- | --- | --- |
| [#6](https://www.ncbi.nlm.nih.gov/pubmed) | #4 NOT #5 | 741 |
| [#5](https://www.ncbi.nlm.nih.gov/pubmed) | "Vietnam Conflict"[MeSH] OR Vietnam War[tiab] OR Military[tiab] OR Asian American*[tiab] OR Vietnamese American*[tiab] OR "Stress Disorders, Post-Traumatic"[MeSH] OR ptsd*[tiab] OR posttraumatic stress disorder*[tiab] OR post traumatic stress disorder*[tiab] | 79202 |
| [#4](https://www.ncbi.nlm.nih.gov/pubmed) | #1 AND #2 AND #3 | 1134 |
| [#3](https://www.ncbi.nlm.nih.gov/pubmed) | "Vietnam"[MeSH] OR vietnam*[tiab] OR viet nam*[tiab] OR vietnam*[ad] OR viet nam*[ad] OR hanoi[tiab] OR hanoi[ad] OR thai nguyen[tiab] OR thai nguyen[ad] OR hue[tiab] OR hue[ad] OR ho chi minh[tiab] OR ho chi minh[ad] | 22558 |
| [#2](https://www.ncbi.nlm.nih.gov/pubmed) | "Cardiovascular Diseases"[Mesh] OR "Chronic Disease"[Mesh] OR "Hypertension"[Mesh:noexp] OR "Comorbidity"[Mesh] OR chronic*[tiab] OR non communicable*[tiab] OR noncommunicable*[tiab] OR cardiovascular*[tiab] OR cvd*[tiab] OR coronary disease*[tiab] OR heart disease*[tiab] OR hypertensi*[tiab] OR htn*[tiab] OR HBP[tiab] OR blood pressure*[tiab] OR comorbidit*[tiab] OR co morbidit*[tiab] OR multimorbidit*[tiab] OR multi morbidit*[tiab] | 3502102 |
| [#1](https://www.ncbi.nlm.nih.gov/pubmed) | "Long-term Care"[MeSH Terms] OR "economics" [Subheading] OR "Delivery of Health Care, Integrated"[Mesh] OR "Health Services"[Mesh] OR "Health Services Accessibility"[MeSH:noexp] OR "Health Services Administration"[MeSH:noexp] OR "Personal Health Services"[MeSH:noexp] OR "Community Health Services"[Mesh:noexp] OR "Health Services Needs and Demand"[Mesh:noexp] OR "Health Care Economics and Organizations"[MeSH] OR "Health Care Quality, Access, and Evaluation"[MeSH] OR "Patient Care Management"[Mesh:noexp] OR "Delivery of Health Care"[Mesh:noexp] OR "Healthcare Disparities"[Mesh] OR "Health Equity"[Mesh] OR "Patient Acceptance of Health Care"[Mesh] OR "Quality of Health Care"[Mesh] OR "Patient Compliance"[Mesh] OR "Health Policy"[Mesh] OR "Utilization"[Subheading] OR "Supply and distribution"[Subheading] OR community[tiab] OR comprehensive health*[tiab] OR primary health*[tiab] OR patient centred[tiab] OR patient centered[tiab] OR patient focus*[tiab] OR model of care[tiab] OR models of care[tiab] OR retention in care[tiab] OR complian*[tiab] OR ((continuity[tiab] OR adher*[tiab] OR integration*[tiab]) AND care[tiab]) OR continuum of care[tiab] OR ((health[tiab] OR healthcare*[tiab]) AND (equit*[tiab] OR inequal*[tiab] OR equal*[tiab] OR intervention*[tiab] OR policy[tiab] OR policies[tiab] OR insurance*[tiab] OR expenditure*[tiab] OR cost[tiab] OR seek*[tiab] OR satisf*[tiab] OR need[tiab] OR needs[tiab] OR needing*[tiab] OR demand*[tiab] OR system*[tiab] OR literacy[tiab] OR service*[tiab] OR integrat*[tiab] OR quality[tiab] OR access[tiab] OR accessibility[tiab] OR utiliz*[tiab] OR utilis*[tiab])) OR "Quality of Life"[Mesh] OR life qualit*[tiab] OR "quality of life"[tiab] OR "Activities of Daily Living"[Mesh] OR "activities of daily living"[tiab] OR daily living activit*[tiab] OR "adl"[tiab] OR "chronic limitation of activity"[tiab] OR self care*[tiab] OR "Health Status"[Mesh] OR "health status"[tiab] OR "level of health"[tiab] OR health level*[tiab] OR "qol"[tiab] OR "hrql"[tiab] OR "hrqol"[tiab] OR economic*[tiab] OR cost effect*[tiab] OR longterm*[tiab] OR long term*[tiab] | 8485137 |

**Embase.com**

| **No.** | **Query** | **6/6/2017** |
| --- | --- | --- |
| #6 | #4 NOT #5 | 993 |
| #5 | 'posttraumatic stress disorder'/exp OR 'vietnam war':ti,ab OR military:ti,ab OR 'asian american*':ti,ab OR 'vietnamese american*':ti,ab OR 'migration'/exp OR ptsd*:ti,ab OR 'posttraumatic stress disorder*':ti,ab OR 'post traumatic stress disorder*':ti,ab | 135621 |
| #4 | #1 AND #2 AND #3 | 1347 |
| #3 | 'viet nam'/exp OR vietnam*:ti,ab OR 'viet nam*':ti,ab OR vietnam*:ca OR 'viet nam*':ca OR hanoi:ti,ab OR hanoi:ca OR 'thai nguyen':ti,ab OR 'thai nguyen':ca OR hue:ti,ab OR hue:ca OR 'ho chi minh':ti,ab OR 'ho chi minh':ca | 23972 |
| #2 | 'cardiovascular disease'/exp OR 'chronic disease'/exp OR 'hypertension'/de OR 'essential hypertension'/exp OR 'borderline hypertension'/exp OR 'hypertensive crisis'/exp OR 'malignant hypertension'/exp OR 'metabolic syndrome x'/exp OR 'prehypertension'/exp OR 'resistant hypertension'/exp OR 'systolic hypertension'/exp OR 'comorbidity'/exp OR chronic*:ti,ab OR 'non communicable*':ti,ab OR noncommunicable*:ti,ab OR cardiovascular*:ti,ab OR cvd*:ti,ab OR 'coronary disease*':ti,ab OR 'heart disease*':ti,ab OR hypertensi*:ti,ab OR htn*:ti,ab OR hbp:ti,ab OR 'blood pressure*':ti,ab OR comorbidit*:ti,ab OR 'co morbidit*':ti,ab OR multimorbidit*:ti,ab OR 'multi morbidit*':ti,ab | 5222133 |
| #1 | 'daily life activity'/exp OR 'quality of life'/exp OR 'economic aspect'/exp OR 'health care delivery'/de OR 'care bundle'/exp OR 'integrated health care system'/exp OR 'long term care'/exp OR 'primary health care'/exp OR 'secondary health care'/exp OR 'self care'/exp OR 'ambulatory care'/exp OR 'health service'/de OR 'family service'/exp OR 'elderly care'/de OR 'patient care planning'/exp OR 'case management'/exp OR 'patient decision making'/exp OR 'blood pressure monitoring'/exp OR 'home monitoring'/exp OR 'self monitoring'/exp OR 'patient referral'/exp OR 'patient scheduling'/exp OR 'patient selection'/exp OR 'rural health care'/exp OR 'health services research'/exp OR 'medical service'/exp OR 'health care quality'/de OR 'health equity'/exp OR 'performance measurement system'/exp OR 'practice guideline'/exp OR 'professional standard'/de OR 'professional standards review organization'/exp OR 'utilization review'/exp OR 'health disparity'/exp OR 'social determinants of health'/exp OR 'patient compliance'/exp OR 'health care system'/exp OR 'health care utilization'/exp OR 'managed care'/exp OR 'medical information system'/exp OR 'medical record'/exp OR 'resource management'/exp OR communit*:ti,ab OR 'comprehensive health*':ti,ab OR 'primary health*':ti,ab OR 'patient centred':ti,ab OR 'patient centered':ti,ab OR 'patient focus*':ti,ab OR 'model of care':ti,ab OR 'models of care':ti,ab OR 'retention in care':ti,ab OR complian*:ti,ab OR (continuity:ti,ab OR adher*:ti,ab OR integration*:ti,ab AND care:ti,ab) OR 'continuum of care':ti,ab OR (health:ti,ab OR healthcare*:ti,ab AND (equit*:ti,ab OR inequal*:ti,ab OR equal*:ti,ab OR intervention*:ti,ab OR policy:ti,ab OR policies:ti,ab OR insurance*:ti,ab OR expenditure*:ti,ab OR cost:ti,ab OR seek*:ti,ab OR satisf*:ti,ab OR need:ti,ab OR needs:ti,ab OR needing*:ti,ab OR demand*:ti,ab OR system*:ti,ab OR literacy:ti,ab OR service*:ti,ab OR integrat*:ti,ab OR quality:ti,ab OR access:ti,ab OR accessibility:ti,ab OR utiliz*:ti,ab OR utilis*:ti,ab)) OR 'life qualit*':ti,ab OR 'quality of life':ti,ab OR 'activities of daily living':ti,ab OR 'daily living activit*':ti,ab OR 'adl':ti,ab OR 'chronic limitation of activity':ti,ab OR 'self care*':ti,ab OR 'health status':ti,ab OR 'level of health':ti,ab OR 'health level*':ti,ab OR qol:ti,ab OR hrql:ti,ab OR hrqol:ti,ab OR economic*:ti,ab OR 'cost effect*':ti,ab OR longterm*:ti,ab OR 'long term*':ti,ab | 7956564 |

**Ebsco/CINAHL**

| **No.** | **Query** | **6/6/2017** |
| --- | --- | --- |
| S26 | S20 NOT S25 | 77 |
| S25 | ( S21 OR S22 OR S23 ) OR TI ( “vietnam war” OR military OR “asian american*” OR “vietnamese american*” OR ptsd* OR “posttraumatic stress disorder*” OR “post traumatic stress disorder*” ) OR AB ( “vietnam war” OR military OR “asian american*” OR “vietnamese american*” OR ptsd* OR “posttraumatic stress disorder*” OR “post traumatic stress disorder*” ) | 30,397 |
| S24 | S21 OR S22 OR S23 | 18,992 |
| S23 | (MH "Vietnam Veterans") | 213 |
| S22 | (MH "Immigrants+") | 8,645 |
| S21 | (MH "Stress Disorders, Post-Traumatic") | 10,260 |
| S20 | S8 AND S15 AND S19 | 158 |
| S19 | S17 OR S18 | 3,349 |
| S18 | (MH "Vietnamese") | 369 |
| S17 | (MH "Vietnam") OR AF ( vietnam* OR “viet nam*” OR hanoi OR “thai nguyen” OR hue OR “ho chi minh” ) OR TI ( vietnam* OR “viet nam*” OR hanoi OR “thai nguyen” OR hue OR “ho chi minh” ) OR AB ( vietnam* OR “viet nam*” OR hanoi OR “thai nguyen” OR hue OR “ho chi minh” ) | 3,273 |
| S16 | (MH "Vietnam") | 1,688 |
| S15 | ( S9 OR S10 OR S11 OR S12 OR S13 ) OR TI ( chronic* OR “non communicable*” OR noncommunicable* OR cardiovascular* OR cvd* OR “coronary disease*” OR “heart disease*” OR hypertensi* OR htn* OR hbp OR “blood pressure*” OR comorbidit* OR “co morbidit*” OR multimorbidit* OR “multi morbidit*” ) OR AB ( chronic* OR “non communicable*” OR noncommunicable* OR cardiovascular* OR cvd* OR “coronary disease*” OR “heart disease*” OR hypertensi* OR htn* OR hbp OR “blood pressure*” OR comorbidit* OR “co morbidit*” OR multimorbidit* OR “multi morbidit*” ) | 267,401 |
| S14 | S9 OR S10 OR S11 OR S12 OR S13 | 90,479 |
| S13 | (MH "Comorbidity") | 27,722 |
| S12 | (MH "Prehypertension") | 73 |
| S11 | (MH "Metabolic Syndrome X") | 5,622 |
| S10 | (MH "Chronic Disease") | 33,566 |
| S9 | (MH "Hypertension") OR (MH "Hypertensive Crisis") OR (MH "Hypertension, Isolated Systolic") OR (MH "Hypertension, Malignant") OR (MH "Hypertension, Refractory") OR (MH "Masked Hypertension") | 28,017 |
| S8 | ( S1 OR S2 OR S3 OR S4 OR S5 OR S6 ) OR TI ( communit* OR “comprehensive health*” OR “primary health*” OR “patient centred” OR “patient centered” OR “patient focus*” OR “model of care” OR “models of care” OR “retention in care” OR complian* OR (continuity OR adher* OR integration* AND care) OR “continuum of care” OR ((health OR healthcare*) AND (equit* OR inequal* OR equal* OR intervention* OR policy OR policies OR insurance* OR expenditure* OR cost OR seek* OR satisf* OR need OR needs OR needing* OR demand* OR system* OR literacy OR service* OR integrat* OR quality OR access OR accessibility OR utiliz* OR utilis*)) OR “life qualit*” OR “quality of life” OR “activities of daily living” OR “daily living activit*” OR “adl” OR “chronic limitation of activity” OR “self care*” OR “health status” OR “level of health” OR “health level*” OR qol OR hrql OR hrqol OR economic* OR “cost effect*” OR longterm* OR “long term*” ) OR AB ( communit* OR “comprehensive health*” OR “primary health*” OR “patient centred” OR “patient centered” OR “patient focus*” OR “model of care” OR “models of care” OR “retention in care” OR complian* OR (continuity OR adher* OR integration* AND care) OR “continuum of care” OR ((health OR healthcare*) AND (equit* OR inequal* OR equal* OR intervention* OR policy OR policies OR insurance* OR expenditure* OR cost OR seek* OR satisf* OR need OR needs OR needing* OR demand* OR system* OR literacy OR service* OR integrat* OR quality OR access OR accessibility OR utiliz* OR utilis*)) OR “life qualit*” OR “quality of life” OR “activities of daily living” OR “daily living activit*” OR “adl” OR “chronic limitation of activity” OR “self care*” OR “health status” OR “level of health” OR “health level*” OR qol OR hrql OR hrqol OR economic* OR “cost effect*” OR longterm* OR “long term*” ) | 1,213,798 |
| S7 | S1 OR S2 OR S3 OR S4 OR S5 OR S6 | 926,527 |
| S6 | (MH "Medical Records") OR (MH "Medical Record Linkage") OR (MH "Medical Records, Personal") OR (MH "Policy and Procedure Manuals") OR (MH "Medical Transcription") | 14,218 |
| S5 | (MH "Compliance Care (Saba CCC)+") OR (MH "Adherence Behavior (Iowa NOC)") OR (MH "Compliance Behavior (Iowa NOC)") | 11 |
| S4 | (MH "Healthcare Disparities") OR (MH "Health Resource Allocation") OR (MH "Health Resource Utilization") | 20,304 |
| S3 | (MH "Social Determinants of Health") OR (MH "Health Status Disparities") | 3,500 |
| S2 | (MH "Quality of Health Care") OR (MH "Guideline Adherence") OR (MH "Practice Guidelines") OR (MH "Process Assessment (Health Care)") OR (MH "Program Evaluation") OR (MH "Quality Improvement") OR (MH "Quality Management, Organizational") OR (MH "Quality Assessment+") OR (MH "Quality Assurance") | 174,341 |
| S1 | (MH "Health Services Needs and Demand+") OR (MH "National Health Programs+") OR (MH "Health Resource Utilization") OR (MH "Health Resource Allocation") OR (MH "Strategic Planning+") OR (MH "Performance Measurement Systems") OR (MH "Quality of Health Care+") OR (MH "Clinical Governance+") OR (MH "Outcomes (Health Care)") OR (MH "Outcome Assessment") OR (MH "Utilization Review+") OR (MH "Quality Management, Organizational") OR (MH "Health Services Research+") OR (MH "Evaluation Research+") OR (MH "Action Research") OR (MH "Descriptive Research") OR (MH "Policy Studies+") OR (MH "Patient Selection") OR (MH "Appointment and Scheduling Information Systems") OR (MH "Practice Management Information Systems") OR (MH "Management Information Systems") OR (MH "Referral (Iowa NIC)") OR (MH "Referral and Consultation") OR (MH "Remote Consultation") OR (MH "Blood Pressure Determination+") OR (MH "Decision Making, Patient+") OR (MH "Decision Making, Organizational") OR (MH "Case Management") OR (MH "Continuity of Patient Care+") OR (MH "Family Centered Care+") OR (MH "Disease Management") OR (MH "Multidisciplinary Care Team") OR (MH "Patient Centered Care") OR (MH "Family Services") OR (MH "Community Health Services") OR (MH "Community Networks") OR (MH "Consumer Participation") OR (MH "Health Services") OR (MH "Ambulatory Care") OR (MH "Community Health Centers") OR (MH "Hospitals") OR (MH "Hospitals, Public+") OR (MH "Hospitals, Federal+") OR (MH "Hospitals, Rural") OR (MH "Hospitals, Urban") OR (MH "Ambulatory Care Facilities") OR (MH "Self Care") OR (MH "Self Care Agency") OR (MH "Self Medication") OR (MH "Long Term Care") OR (MH "Cardiovascular Care") OR (MH "Holistic Care") OR (MH "Medical Care") OR (MH "Patient Care") OR (MH "Protocols+") OR (MH "Patient Care Plans") OR (MH "Health Care Delivery") OR (MH "Health Care Reform+") OR (MH "Health Services Accessibility+") OR (MH "National Health Programs") OR (MH "Primary Health Care") OR (MH "Secondary Health Care") OR (MH "Health Resource Allocation") OR (MH "Health Resource Utilization") OR (MH "Healthcare Disparities") OR (MH "Health Care Delivery, Integrated") OR (MH "Resource Allocation+") OR (MH "Fees and Charges+") OR (MH "Financing, Organized") OR (MH "Financing, Government+") OR (MH "Public Assistance+") OR (MH "Insurance, Disability+") OR (MH "Insurance, Health+") OR (MH "Insurance+") OR (MH "Managed Care Programs+") OR (MH "Insurance Selection Bias+") OR (MH "Insurance, Health, Reimbursement+") OR (MH "Reimbursement Mechanisms+") OR (MH "Prospective Payment System+") OR (MH "Fund Raising") OR (MH "Budgets") OR (MH "Health Services Purchasing+") OR (MH "Medical Savings Accounts") OR (MH "Risk Management") OR (MH "Health Care Costs+") OR (MH "Diagnosis-Related Groups+") OR (MH "Cost Control+") OR (MH "Costs and Cost Analysis") OR (MH "Quality of Life+") OR (MH "Quality of Life (Iowa NOC)") | 917,889 |

**Thomson Reuters/Web of Science 28/November/2016**

| **No.** | **Query** | **6/6/2017** |
| --- | --- | --- |
| #6 | #4 NOT #5  *Indexes=SCI-EXPANDED, SSCI, A&HCI, ESCI Timespan=All years* | 469 |
| #5 | TOPIC=(“vietnam war” OR military OR “asian american*” OR “vietnamese american*” OR ptsd* OR “posttraumatic stress disorder*” OR “post traumatic stress disorder*”)  *Indexes=SCI-EXPANDED, SSCI, A&HCI, ESCI Timespan=All years* | 128,712 |
| #4 | #3 AND #2 AND #1  *Indexes=SCI-EXPANDED, SSCI, A&HCI, ESCI Timespan=All years* | 1,056 |
| #3 | TOPIC=(vietnam* OR “viet nam*” OR hanoi OR “thai nguyen” OR hue OR “ho chi minh”)  *Indexes=SCI-EXPANDED, SSCI, A&HCI, ESCI Timespan=All years* | 47,268 |
| #2 | TOPIC=(chronic* OR “non communicable*” OR noncommunicable* OR cardiovascular* OR cvd* OR “coronary disease*” OR “heart disease*” OR hypertensi* OR htn* OR hbp OR “blood pressure*” OR comorbidit* OR “co morbidit*” OR multimorbidit* OR “multi morbidit*”)  *Indexes=SCI-EXPANDED, SSCI, A&HCI, ESCI Timespan=All years* | 2,041,975 |
| #1 | TOPIC=(communit* OR “comprehensive health*” OR “primary health*” OR “patient centred” OR “patient centered” OR “patient focus*” OR “model of care” OR “models of care” OR “retention in care” OR complian* OR (continuity OR adher* OR integration* AND care) OR “continuum of care” OR ((health OR healthcare*) AND (equit* OR inequal* OR equal* OR intervention* OR policy OR policies OR insurance* OR expenditure* OR cost OR seek* OR satisf* OR need OR needs OR needing* OR demand* OR system* OR literacy OR service* OR integrat* OR quality OR access OR accessibility OR utiliz* OR utilis*)) OR “life qualit*” OR “quality of life” OR “activities of daily living” OR “daily living activit*” OR “adl” OR “chronic limitation of activity” OR “self care*” OR “health status” OR “level of health” OR “health level*” OR qol OR hrql OR hrqol OR economic* OR “cost effect*” OR longterm* OR “long term*”)  *Indexes=SCI-EXPANDED, SSCI, A&HCI, ESCI Timespan=All years* | 4,397,833 |
