# Supplementary material for: Access to hypertension care and services in primary health-care settings in Vietnam: a systematic narrative review of existing literature
Source: Glob Health Action. 2019 May 23;12(1):1610253. doi: 10.1080/16549716.2019.1610253 (PMC6534204; doi:10.1080/16549716.2019.1610253)
Supplement: Supplemental Material [file ZGHA_A_1610253_SM1717.zip › S Table 2.docx]

**Overview of reported training projects related to hypertension care in Vietnam**

| **Purpose/Design** | **Main Results** | **Recommended Next Steps** |
| --- | --- | --- |
| **Trainings organized by National Hypertension Program** | | |
| *JAHR, 2014* | | |
| The training for commune health workers focused on 1) measuring blood pressure, 2) methods for changing lifestyles to prevent the disease, 3) drug treatment for hypertension, and 4) models for prevention, treatment, and management of hypertension in the community | - By the end of 2013, the project organised 2,476 training sessions with 71,278 health workers. | - Continuation of training programs |
| **Train healthcare practitioners on adding physical activity on prescription** | | |
| *Sundberg, 2012* | | |
| This was a Swedish aid agency funded project, included following components:  1) Workshops on “Promoting Physical Activities in Non-communicable Diseases (NCDs) Prevention”,  2) Study visits to Sweden,  3) Selective translation of a handbook of physical activity (PA) prescription from English to Vietnamese,  4) Development of curriculum and training material,  5) Training of trainers (TOT) courses in Sweden,  6) Training courses (partly case-based) for health care practitioners in Vietnam,  7) Mass media campaign,  8) Evaluation of effectiveness after training for patients and health care providers,  9) International Conference in Hanoi in November 2012, and  10) Process to help introduce National Guidelines on PA for NCDs Prevention. | - 120 healthcare practitioners were trained; - Based on the feedback from 50 of them, the practitioners mentioned:  1) Have a markedly greater understanding of the relation between life style and disease;  2) Have made implementation plans in their own clinical setting. | - Structured training of healthcare practitioners in Vietnam can increase interest, knowledge and willingness to change clinical practice in the field of physical activity on prescription |
| **Train family medicine specialists posted at primary healthcare level facilities** | | |
| *Islam, 2014* | | |
| Initiative at the Department of Family Medicine, Hue University to train family medicine and paramedical personnel posted to primary healthcare level facilities and communities in order to meet the increasing demand, and increase the availability of human resources in health care considerably | - Doctors completing 6 years of medical training do currently an additional 2-year curriculum in family medicine; - About 100 doctors have graduated as family medicine specialists in Vietnam. | - A scaling up of the training program to all provinces of Vietnam and a national proposal to reduce overloading at hospitals is on-going (2012–2020). |
| *Markuns, 2015* | | |
| Primary efforts have targeted capacity building through training of a competent primary care workforce, initially focused on developing formal specialist training for primary care physicians.  Funding: Current funding from the China Medical Board, the Atlantic Philanthropies, and GHETS. | - Over 600 family physician graduates and continued training at most universities. - Quantitative evaluation results have shown that knowledge and confidence of physicians are improved in multiple clinical areas, and measured observations of clinical practice indicate improvements. - Ministries of Health and local health authorities are highly supportive of these programs and continue to seek enrolment of their health staff in these programs. - Key to the sustainability of this approach has been the engagement of local stakeholders for policy integration coupled with implementation performed exclusively by local partner universities to train and support primary care physicians. | - Maintaining training capacity and carrying out national-level scale-up remain the biggest challenges. - The World Bank has recently entered into a $126 million Health Professionals Education and Training for Health System Reforms Project with Vietnam. |
