# Supplementary material for: Access to hypertension care and services in primary health-care settings in Vietnam: a systematic narrative review of existing literature
Source: Glob Health Action. 2019 May 23;12(1):1610253. doi: 10.1080/16549716.2019.1610253 (PMC6534204; doi:10.1080/16549716.2019.1610253)
Supplement: Supplemental Material [file ZGHA_A_1610253_SM1717.zip › S Table 3.docx]

**Overview of reported community-based and therapeutic interventions related to hypertension care in Vietnam**

| **Intervention** | **Main Results/Conclusions** |
| --- | --- |
| **Hypertension Control** |  |
| Community-based hypertension management program integrated with a community-targeted healthy lifestyle promotion with two components (*Nguyen, 2011; Nguyen, 2012; Lim, 2014)*:   - Managing hypertensive people at the commune health station - Top-down approach: involvement of local healthcare team and authorities in all screening activities, and to train and build up a local cardiac care team supported by standardized workflows, manuals, guidelines, forms and essential drug stocks for hypertension and supervised by higher level doctors; - Delivering health education on cardiovascular diseases risk factors to the entire community. - Bottom-up approach: core information, communication, and education (ICE) messages were broadcast repeatedly to improve local awareness and encourage lifelong check-ups and treatment. Monitor blood pressure and inform patients about the change to improve their adherence. | - The program became independent after 17 months; - The success of the program is contributed by strategic utilization of human capital through:   1) successful engagement of the whole community;  2) support from committed local authorities and medical expertise; and  3) training of committed health care workers;   - Comparing intervention commune with a reference commune, - There was a significant reduction in systolic and diastolic blood pressure in the general population at the intervention commune. - It also reduced levels of salty diet; - There was limited effect on daily smoking and heavy alcohol consumption. |
| Two community-based interventions to promote engagement in the care of persons diagnosed with hypertension in rural Vietnam (*Nguyen, 2017)*:   - Storytelling intervention: Two interactive DVDs, each with five stories for patients speaking in Vietnamese about the health consequences of hypertension, overcoming barriers to hypertension control and the importance of adherence to prescribed medication, quitting smoking, dietary changes, weight loss, reducing sodium/salt intake, increasing levels of physical activity, and moderate use of alcohol. Each DVD was approximately 50 min in length; - Didactic intervention: The DVD included general recommendations for controlling non-communicable diseases (NCDs) including the importance of having a healthy diet, participation in regular physical exercise, quitting smoking tobacco and drinking less or no alcohol, and need for regular examination check-ups; in addition to information about other NCDs other than hypertensives. | - The study was not specifically powered to detect statically significant differential changes over time; - However, mean systolic and diastolic blood pressure readings improved over time for both study groups, and the reduction was greater in storytelling vs. didactic intervention groups; - Self-reported blood pressure medication adherence rates improved for storytelling intervention group but worsened for the didactic intervention group. |
| **NCDs Prevention & Control** |  |
| Free temporary clinics as part of a charity’s medical program (*Barzin, 2012):*   - Provides free temporary medical services for rural populations in central Vietnam - Charity developed the program under request of local authorities. | - Majority of diagnoses were for chronic problems which the program is not designed to manage; - Clinics duplicate existing services and do not contribute to development of the public health system. |
| Tai Chi training and its effect on blood pressure in elderly (*Nguyen, 2012):*  Target population was elderly in general, not only hypertensive patients. | - Tai Chi group showed better results than the control group for blood pressure at three test periods; the effects of Tai Chi training remained in physical performance levels and subjectively perceived health as well as blood pressure for at least 8 weeks after stopping Tai Chi training. |
| Community-based intervention; Communications for Behavioural Impact-Eat Less Salt Intervention (COMBI-ELS). Integrating five communication action areas (*Do, 2016)*: 1) Public advocacy to mobilize decision makers; 2) Community mobilization to engage community leaders; 3) Sustained community-based social marketing to remind communities of the problems; 4) Information provision to individuals through face-to-face engagement; and 5) Point-of-service promotion to services such as healthcare centres.  Target population was adults 25-94yr, not only hypertensive patients, living in selected communes and wards of Viet Tri city. | - About twice as many people were aware that high salt intake could cause hypertension (43.61 vs. 86.30%, P-value <.001); - The mean systolic and diastolic blood pressure were significantly lower following the intervention; the proportion of respondents with hypertension was 26.13% (n=133) at baseline and 21.14% (n=108) at follow up, not statistically significant; - Among those with hypertension, the proportion of participants who were aware of their hypertensives status was significantly higher at follow up (50.93%) compared with baseline (34.59%); additionally, the proportion of hypertensive subjects with no management or treatment was significantly lower at follow up (56.48%) compared with baseline (70.68%). |
