# Supplementary material for: Access to hypertension care and services in primary health-care settings in Vietnam: a systematic narrative review of existing literature
Source: Glob Health Action. 2019 May 23;12(1):1610253. doi: 10.1080/16549716.2019.1610253 (PMC6534204; doi:10.1080/16549716.2019.1610253)
Supplement: Supplemental Material [file ZGHA_A_1610253_SM1717.zip › S Table 4.docx]

**Overview of studies that investigated adherence to treatment related to hypertension care in Vietnam**

| **Adherence Level/s** | **Associations** |
| --- | --- |
| *Nguyen QN, 2011*  **Compliance definition:** Number of check-ups (clinic visits) for each participant during the 17mo follow-up period | |
| - Regular follow-up or 1 check-up per 1/2 months (n=326, 65.6%) - Irregular follow-up or 1 check-up per 3/6 months (n=100, 20.1%) - Drop-out or >1 check-up per 6 months (n=71, 14.3%) | - Associations with drop out:   - Age, younger people: more likely to drop out   - Mild/less severe hypertension initially: significantly more likely to drop out   - Have cardiovascular disease history: less likely to drop out   - Manual labours: more likely to drop out - Consequences for follow up groups:   - Showed significant reductions of systolic and diastolic blood pressure;   - They received more combined drugs or higher doses - Independent predictors for programme compliance; adjusting to age, sex, % of systolic and diastolic blood pressure changes, # of behavioural risk factors, having cardiovascular disease history, having chronic disease history, having family history for cardiovascular disease, number of tablets to take daily, number of drugs to combine and having a minor event:   - Changes in blood pressure (either systolic or diastolic blood pressure);   - Number of combined antihypertensive drugs. |
| *Ha, 2014*  **Adherence definition:** Self-reported attendance to follow-up appointments and taking medication appropriately | |
| Adherent (n=147/275, 53.5%) | - Associations with adherence to treatment:   - Higher mean of score for four quality of life (QOL)'s domains - Consequences for adherence to treatment:   - Significantly increased scores for three QOL's domains (i.e. physical health, psychological health & environment) |
| *Nguyen TPL, 2017*  **Adherence definition:** Number of pill-days covered: the number of days the drug was taken divided by the total number of days since the first day of prescribing during a one-year follow-up period | |
| Quantitative data: Adherence/non-adherence is differentiated at the threshold of 80%  Adherent (n=157/315, 49.8%) | - Associations with adherence to treatment; after adjusting for sex and cardiovascular disease risk level:   - Age: each 1-year increase in age resulted in subjects being 1.04 times more likely to be adherent (95% Confidence Interval: 1.00-1.07; P-value=0.04) |
| Qualitative data: In-depth interviews with 18 patients; patients were asked about reasons for adherence and non-adherence.   - Change in classification in quantitative data   Of adherents (n=11) -> 2 non-adherents  Of non-adherents (n=7) -> 4 adherents   - After change: adherent (n=13), and non-adherent (n=5) | - Factors related to adherence   - Be aware of complications   - Have a family experience with complications - Factors related to non-adherence   - Forget/busy,   - Stop medications after feeling better,   - Have side effects |
| *Nguyen HL, 2017*  **Adherence definition:** Used an adapted eight-item scale for Vietnamese patients with hypertension, with greater scores indicating worse adherence. | |
| Moderate to high adherence (i.e. 0-2 points);  At baseline After 3 months  Storytelling: 62.3% 77.5%  Didactic: 72.8% 57.4% | - One patients said: “after watching the DVD, I asked my grandson to set up the alarm in my cell phone to remind me to take medications on time even if I am in the middle of doing something”. - Possible information bias (self-reported medication adherence) and placebo effect |
