# Supplementary material for: Access to hypertension care and services in primary health-care settings in Vietnam: a systematic narrative review of existing literature
Source: Glob Health Action. 2019 May 23;12(1):1610253. doi: 10.1080/16549716.2019.1610253 (PMC6534204; doi:10.1080/16549716.2019.1610253)
Supplement: Supplemental Material [file ZGHA_A_1610253_SM1717.zip › S Table 5.docx]

**Overview of studies that investigated quality of life related to hypertensive patients in Vietnam**

| **Sample** | **Instrument** | **Scores** | **Significant predictors** | |
| --- | --- | --- | --- | --- |
|  |  |  | **Higher scores (positive association)** | **Lower scores (negative association)** |
| **Hypertension Patients** | | | | |
| *Ha, 2014* | | | | |
| n=275  Age ≥50yr  Random sampling of list of patients at commune health stations of a rural commune, Long An Province | WHOQOL-BREF Questionnaire (26 items)  Score: 0-100  4 domains   - Physical health - Psychological health - Social relationships - Environmental health | Overall average: 56.93  Mean score by domain:   - Physical health, 54.7±14.9 - Psychological health, 49.4±12.7 - Social relationships, 64.1±14.1 - Environmental health, 59.5±10.4 | - Being male: for psychological health; - Married: for all domains; - Attainment of higher education: for environmental health; - Having physical activity at a moderate level: for all domains; - Adherence to treatment: for physical, psychological health & social relationships. | - older age: for physical, psychological health; - presence of co-morbidity: for psychological health. |
| *Nguyen TPL, 2015* | | | | |
| n=722 (691 with complete records)  Age 41-80yr  Recruited at outpatient clinic in Thai Nguyen General Hospital | Quality Metric's Short-form 36v2 (11 items)  Score: 0-1  6 domains   - Physical domain - Role domain - Social domain - Pain domain - Mental domain - Vitality domain | Mean score for health-state utility: 0.73±0.14 | - Being male. | - Patients 70-80yr old compared to younger groups; - Patients with more than three comorbidities compared to patients with no comorbidity. |
| **Elderly** | | | | |
| *Nguyen MH, 2012* | | | | |
| Intervention with 6-month of Tai Chi Training.  Intervention group: n=48  Control group: n=48  Community-dwelling, age 60-79yr | Short Form-36  8 domains   - Physical functioning - Role-physical - Bodily pain - General health - Vitality domain - Social functioning - Role-emotional - Mental health | Mean score for SF-36;   - At baseline, At end-test, At follow up,   Tai Chi: 52.31±22.42 80.18±6.56 81.36±5.19  Control: 48.69±23.40 47.97±24.52  P-vale=0.442 <0.000 | | |
